# Automated Feature Mining for Two-Dimensional Liquid Chromatography Applied to Polymers Enabled by Mass Remainder Analysis

**DOI:** 10.1021/acs.analchem.1c05336

**Published:** 2022-03-28

**Authors:** Stef R.A. Molenaar, Bram van de Put, Jessica S. Desport, Saer Samanipour, Ron A.H. Peters, Bob W.J. Pirok

**Affiliations:** † Van ‘t Hoff Institute for Molecular Sciences (HIMS), Analytical Chemistry Group, University of Amsterdam, Science Park 904, Amsterdam 1098 XH, The Netherlands; ‡ Centre for Analytical Sciences Amsterdam (CASA), 1098 XH, Amsterdam, The Netherlands; § TI-COAST, Science Park 904, Amsterdam 1098 XH, The Netherlands; ∥ Covestro, Group Innovation, Physics and Material Science, Waalwijk 5145 PE, The Netherlands

## Abstract

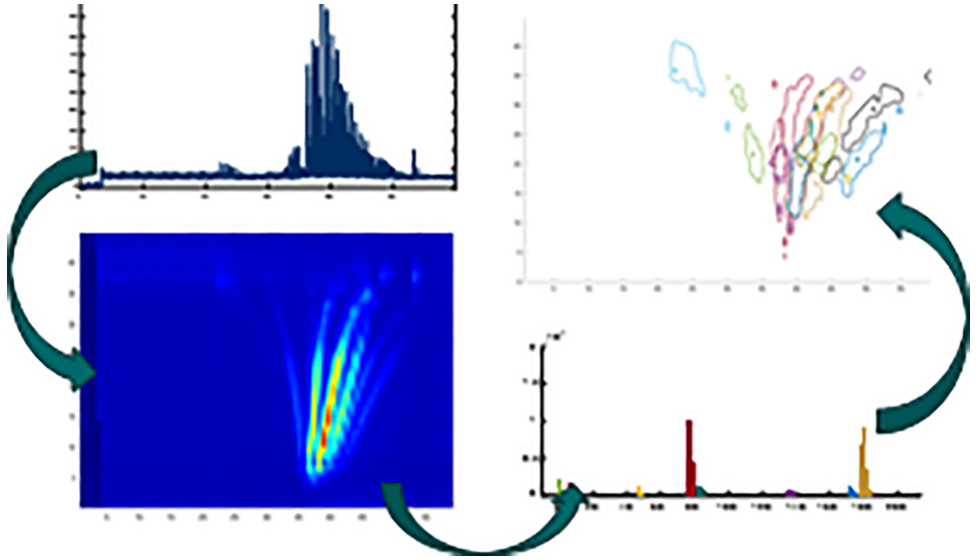

A fast algorithm for automated feature mining of synthetic (industrial) homopolymers or perfectly alternating copolymers was developed. Comprehensive two-dimensional liquid chromatography–mass spectrometry data (LC × LC–MS) was utilized, undergoing four distinct parts within the algorithm. Initially, the data is reduced by selecting regions of interest within the data. Then, all regions of interest are clustered on the time and mass-to-charge domain to obtain isotopic distributions. Afterward, single-value clusters and background signals are removed from the data structure. In the second part of the algorithm, the isotopic distributions are employed to define the charge state of the polymeric units and the charge-state reduced masses of the units are calculated. In the third part, the mass of the repeating unit (*i.e.*, the monomer) is automatically selected by comparing all mass differences within the data structure. Using the mass of the repeating unit, mass remainder analysis can be performed on the data. This results in groups sharing the same end-group compositions. Lastly, combining information from the clustering step in the first part and the mass remainder analysis results in the creation of compositional series, which are mapped on the chromatogram. Series with similar chromatographic behavior are separated in the mass-remainder domain, whereas series with an overlapping mass remainder are separated in the chromatographic domain. These series were extracted within a calculation time of 3 min. The false positives were then assessed within a reasonable time. The algorithm is verified with LC × LC–MS data of an industrial hexahydrophthalic anhydride-derivatized propylene glycol-terephthalic acid copolyester. Afterward, a chemical structure proposal has been made for each compositional series found within the data.

## 1 Introduction

Accurate characterization of polymeric samples is at the core of soft material development as elucidating the structure–property relationships of a given polymer is only possible with a good understanding of a sample’s composition at the molecular level.^1,2^ Within this context, the field of analytical chemistry has been making concerted efforts to develop reliable, multi-dimensional approaches, with comprehensive two-dimensional liquid chromatography (LC × LC) being key.^3−5^ This is because LC × LC allows the simultaneous characterization of multiple molecular characteristics, from determining the molecular weight distribution and chemical composition distribution to functionality and topology distributions.^6^

Applying multi-dimensional characterization often takes the form of combining targeted complimentary techniques; a typical example is the coupling of chromatographic separation(s) with (high-resolution (HR)) mass spectrometry (MS).^7,8^ While chromatography allows separation of the compounds, MS enables their chemical identification. One of the bottlenecks of these modern multi-dimensional methods is the interpretation of the generated data.^9^ The wide variety of instruments deployed for this purpose (*i.e.*, instrument type and manufacturer), together with the incompatibility of their respective data formats, severely inhibits the potential impact of multi-dimensional datasets. Consequently, there is a strong demand for bridging datasets of multi-dimensional analysis in a user-friendly and automated fashion.

At present, the interpretation of LC × LC–MS data starts with the MS dimension and involves molecular formula (MF) assignments. This step is time-consuming since polymer MS spectra are typically complex in terms of information density. Furthermore, polymeric samples are fundamentally unique in that they consist of a mixture of related molecules, each of which differing by one repeating unit, composing a polymer distribution. A given polymer distribution exhibits a distinct characteristic, for instance, a specific end-group, a given chemical composition range, or a peculiar topology. A sample may consist of multiple polymer distributions.

Strategies for the identification of chemically related compounds have been explored and successfully applied to polymer MS analysis. Notably, this includes the so-called Kendrick mass defect (KMD) concept^10,11^ and mass remainder analysis (MARA).^12,13^ While KMD is based on a mass rescaling,^14^ by setting the mass of a monomer unit to an integer, the latter does not require a transformation of the mass domain. Instead, MARA is based on an iterative division of the masses by the exact mass of the repeating unit until no further divisions can be made. The resulting mass remainder (MR) thus embodies information related to chemical composition. It may be noted that similarly to MARA, remainders of Kendrick mass are suitable for identifying homologous series,^15^ as emphasized in a series of comments.^16,17^ In addition, open-access programs have been developed to facilitate polymer MS data treatment, including functionalities for assisted MF assignment, post-calibration, and determination of chemical compositions.^18−20^ Nonetheless, all of the available analytical software packages only address one dimensional datasets and fail to facilitate a broad range of applications.

In this work, we repurposed the MARA approach to methodically reveal data features obtained from an LC × LC–HRMS polymer analysis, and we present an algorithm that was developed to treat the third-order data structure: *t*_R_^1D^, *t*_R_^2D^, *m*/*z*, and I. These are the retention time in chromatographic dimensions 1 and 2, the mass-to-charge ratio, and the intensity of the signal at each retention time and *m*/*z* value, respectively*.* Individual components of the mixture were interrelated by their two-dimensional retention times and MR, which contain chemical composition information. The sample we case-studied consisted of an industrially modified polyester, from which the repeating unit was automatically retrieved by the algorithm. Ultimately, 10, partially separated, polymer distributions and two distributions that underwent sodium exchange of relative abundances as little as 0.6% were identified in a 3 min calculation time and 5 min of manual interpretation of the results.

## 2 Experimental Section

### 2.1 Data Acquisition

The raw LC × LC–HRMS data was acquired from Groeneveld et al.^21^

#### 2.1.1 Chemicals and Samples

The solvents used included *n*-hexane (>99.5%, HiPerSolv grade) and dichloromethane (DCM, >99.8%, HiPerSolv grade) obtained from VWR International (Fontenay-sous-Bois, France). Tetrahydrofuran (unstabilized, GPC grade) was obtained from Biosolve (Valkenswaard, The Netherlands). For mass spectrometry, sodium iodide was used as the ionization agent (>99.5%) and 3-nitrobenzyl alcohol (>99.5%, mass spectrometry grade) was used as the supercharging agent, both obtained from Sigma-Aldrich (Darmstadt, Germany).

The model sample consists of a propylene glycol (PG)–terephthalic acid (TPA) copolyester, which was derivatized with hexahydrophthalic anhydride (HHPA) provided by Covestro (Waalwijk/Zwolle, The Netherlands). The number average molecular weight is estimated to be 1880 Da, based on SEC analysis using polystyrene as the molecular weight calibration. The sample was prepared at a concentration of 20 mg·mL^–1^ in dichloromethane.

#### 2.1.2 Instruments

Two-dimensional LC × LC–HRMS experiments were performed using an Agilent 1290 Infinity 2D- LC system (Agilent Technologies, Waldbronn, Germany) coupled with a Waters Synapt-G2 high-resolution mass spectrometer. The system comprised two binary pumps (G4220A) for solvent delivery, an autosampler (G4226A), column thermostat (G1316C) equipped with a 2D-LC 8-port 2-postion modulation valve (G4236A) with 40 μL loops, and a diode-array detector (G4212A) equipped with an Agilent Max-Light cartridge flow cell (G4212–6008, 10 mm, *V*_det_ = 1.0 μL). The ^1^D column was a Phenomenex Luna HILIC (150 × 2.0 mm i.d., 3.0 μm particles, 200 Å pore size) column used for gradient-NPLC, while two Waters Acquity APC XT columns (75 × 4.6 mm i.d., 1.7 μm particles, 45 Å pore size and 75 × 4.6 mm i.d., 2.5 μm particles, 125 Å pore size, respectively) were coupled in series for SEC experiments.

For parallel UV/HRMS detection, the analytical effluent was split after the second-dimension SEC column set using a tee piece and in-house-made restriction capillaries (450 × 0.075 mm i.d. and 900 × 0.050 mm i.d. capillaries), ensuring a split ratio of 9:1 to the diode array and mass spectrometer, respectively. Using the diverter valve of the Synapt-G2 system, the smallest split flow was combined with a make-up flow (1:1 ratio) consisting of 1 mM NaI with 0.5% (v/v) 3-nitrobenzyl alcohol in deionized water.

#### 2.1.3 Analytical Conditions

The gradient-NPLC ^1^D was thermostated at 23 °C, and a flowrate of 40 μL·min^–1^ was applied with a gradient of 30% (v/v) dichloromethane in hexane (mobile phase A) to 5% (v/v) THF in dichloromethane (mobile phase B). The used gradient program is 0.0–37.5–45.0–46.0–65.0 min 0.0–100.0–100.0–0.0–0.0% B. Between 55.0 and 60.0 min, the flowrate was increased to 0.08 mL·min^–1^ to re-equilibrate the column. The modulation time was set to 45 s, corresponding to a modulation volume of 30 μL and 75% loop filling. The ^2^D SEC separation was operated at 50 °C and run isocratically with THF containing 0.1% (v/v) formic acid using a flowrate of 1.1 mL·min^–1^. The diode-array detectors recorded full spectra from 240 to 400 nm and channels with a specific wavelength of 254 and 262 nm with a bandwidth of 4.8 nm with a scan rate of 40 Hz. Conditions used for the mass spectrometer were as follows: *m*/*z* range, 300–3000; scan rate, 0.2 s; positive ESI; time-of-flight MS resolution mode; capillary voltage, 3.0 kV; sampling cone, 100 V; trap collision voltage, 15 V; source temperature 100 °C; desolvation temperature, 250 °C; nitrogen desolvation gas flow, 800 L·h^–1^; nebulizer gas flow, 100 L·h^–1^. Internal mass calibration was performed using leucine enkephalin as the reference mass.

### 2.2 Data Processing

The entire algorithm was written using MATLAB 2019b (Mathworks, Natick, MA, USA). Raw LC × LC–HRMS data were converted into mzXML format by ProteoWizard 3.0.19202 64-bit.^22^Table 1 shows the user-defined parameters needed for the algorithm. Further explanation of the algorithm is provided in Section 3. A flowchart illustrating a detailed workflow including each user-defined parameter can be found in Supporting Information Section S-1. The algorithm has been incorporated in the open-access MOREDISTRIBUTIONS software.^23^

**Table 1:** User-Defined Parameters Used in the Algorithm

Symbol	Parameter	Value
*I*_min,dp_	ROI analysis: minimum mass peak intensity	100 counts
Δ*m*/*z*_max_	ROI analysis: mass tolerance	0.15 Da
*N*_min,dp_	ROI analysis: minimum number of consecutive datapoints	6 scans
*N*_max,bg_||*I*_min,bg_	background removal: occurrence of signals (*a*) above *b* percent of the maximum ROI intensity	*a* = 2%, *b* = 20%
*d*_Mah1_ || *d*_Mah2_	clustering: maximum Mahalanobis distance	0.05 || 0.15
*M*_rep,min_	MARA: minimum mass of repeat unit	12.0000 Da
ΔMR_max_	MARA: mass remainder tolerance when binning	0.05 Da
*m*_add_	MARA: optional parameter. The mass of the adduct	22.9898 Da

## 3 Results and Discussion

**Figure 1:**
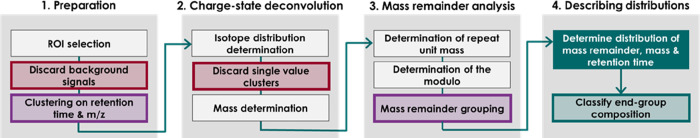
Overview of the proposed data analysis strategy. Colors indicate (red) discard irrelevant data, (purple) grouping of data, and (green) classification of the compositional series.

The data analysis is divided in four main steps: (i) preparation of the data structure, (ii) charge-state deconvolution, (iii) MARA, and (iv) description of the molecular distributions within the polymeric sample. Figure 1 shows an overview of the smaller steps within this strategy, which will be described in further detail below. For a more detailed flowchart, please refer to Supporting Information Section S-1. Note that the original datapoints are not removed during any of these steps so that, after feature mining, additional observations about the identified polymeric series, *e.g.*, size distribution, can be made.

**Figure 2:**
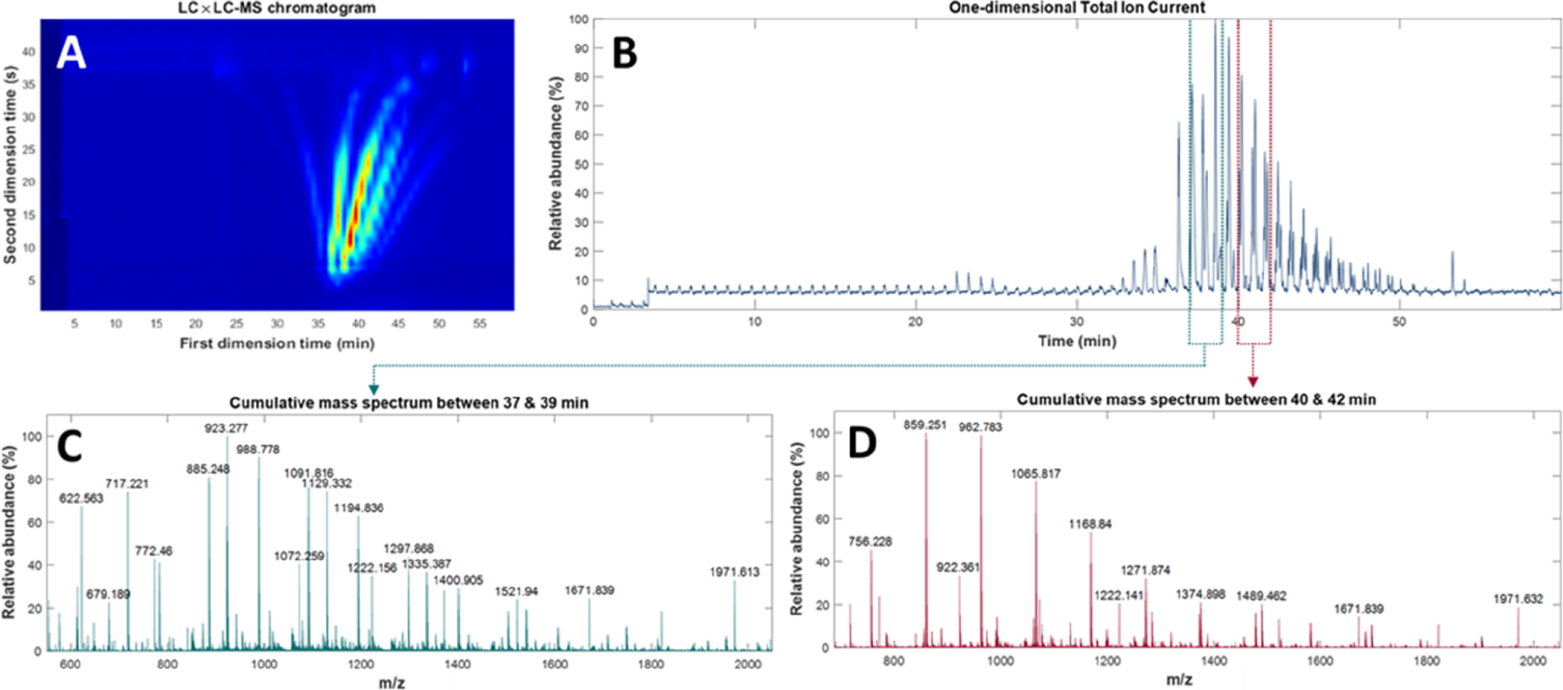
(A) LC × LC–MS plot. (B) Unfolded TIC signal. (C) Cumulative mass spectrum between 37 and 39 min. (D) Cumulative mass spectrum between 40 and 42 min.

Figure 2 presents the raw data used to assess the validity of the developed algorithm. It consists of a total ion current (TIC) chromatogram of 60 min, composed of 14,300 MS spectra, each of which was acquired from *m*/*z* 300 to 3000. The modified polyester exhibits a range of end-groups that are intended to be separated in the first dimension by normal phase chromatography (NPLC), while molecular-weight based separation is addressed in the second dimension via size exclusion chromatography (SEC). The added value of the MS dimension is strongly dependent on the operator’s effort to carefully assign MS peaks and relate their retention times. In practice, an operator will look at the MS spectra at different retention times (often randomly selected) or as the sum of the spectra within a given range. During the investigation of these spectra, structures are assigned to the *m*/*z* with the highest abundance. From these first assignments, a list of theoretical *m*/*z* values is calculated with which extracted ion chromatograms for each degree of polymerization are generated. During this laborious process, secondary distributions of lower abundance and/or unexpected series may be discarded, failing to provide a comprehensive picture of the sample. Although it may be argued that the most abundant polymeric distributions are the focus of an analysis, it can be acknowledged that in many instances, secondary products are the key to better understand the properties of a sample.^24^ Yet, species that are unexpected and have a low relative abundance are the least likely to be identified with manual data processing. The present algorithm aims at overcoming this drawback.

### 3.1 Extracting Information by Region of Interest Analysis and Filtering Unnecessary Data

The algorithm includes a series of user-defined parameters whose values are to be set on an individual basis. These parameters are summarized in Table 1 and may be adjusted based on the kind of mass spectrometer used and the type of LC separation achieved, which ultimately includes the scan rate, the mass resolution and mass accuracy of the data, and the resolution of the chromatographic dimension. Most of these variables play an important role in the first step of the algorithm operation, which aims at preparing the data structure. The region of interest (ROI) analysis^25^ was used to extract points with a minimum intensity (*I*_min_) in the *m*/*z* domain. The LC separation prior to MS analysis allows the exclusion of (random) noise by considering ROIs only if the datapoints within a certain mass tolerance (*m*/*z* ± Δ *m*/*z*) are being found in a given number of successive scans. Provided the peak width of the LC separation here and the used scan rate (0.2 s), the minimum number of consecutive points (*N*_min,dp_) was set to 6.

Background signals, *e.g.*, solvent ions or salt clusters, represent a large amount of the total intensity and are likely to be selected during the ROI analysis. Before appropriate identification of the polymeric structures can be performed, these background signals need to be removed from the data structure. However, the fact that such signals are present continuously allows them to be distinguished from sample-related components. This can be performed automatically by counting the number of datapoints that exceed a set threshold and comparing this with the typical number of datapoints for a chromatographic peak. In this work, an intensity threshold (*I*_min,bg_) of 20% of the maximum ROI intensity has been used. If this threshold was passed in more than 2% of all datapoints (*N*_max,bg_), then the ROI was deemed a background signal and deleted from the data structure. This threshold seemed sufficiently high that real chromatographic peaks are not filtered out. An example of this can be found in Supporting Information S-2.

MARA performs deisotoping of the distribution due to overlap between different polymeric units and their different isotope distributions.^12^ A fundamental difference between MARA and this work is that the isotopic distribution within the sample is utilized. As there is more information available within the dataset (*i.e.*, the chromatographic information), the relative isotopic intensities are not needed to distinguish between these different species. To do this, hierarchical cluster analysis^26^ with a Mahalanobis distance metric^27,28^ (*d*_Mah1_ = 0.05) within both time domains and *m*/*z* domain was employed to define ROI clusters.

In most cases, the differences in *m*/*z* between the isotopes of a compound are significantly smaller than the differences in *m*/*z* between different compounds. The difference in *m*/*z* between isotopes is directly related to the difference in the mass of a ^12^C and a ^13^C atom (1.0033 Da)^29^ divided by the charge (*z*). The benefit of a Mahalanobis metric is that the metric normalizes all dimensions, making differences in clustering ranges (*i.e.*, the time and *m*/*z* ranges) obsolete. Therefore, even if there is mass overlap between different monomer compositions and isotopic distributions, the difference in the time domain is in most instances sufficient to distinguish between different monomeric compositions. In the rare cases where this is not applicable, the next steps of the algorithm filter these datapoints out as explained in Section 3.4.

### 3.2 Charge-State Reduction

Online coupling of liquid separations with mass spectrometry typically involves ionization techniques such as electrospray ionization (ESI). A distinct consequence of ESI usage is the generation of multiple charged ions, resulting in molecules with the same composition existing as various ionic forms [M + *z* × *m*_add_]^±z^, with *z* ≥ 1. The differences in *m*/*z* within each cluster can be exploited to define the charge-state of the polymeric unit. Charge-state deconvolution transforms the signal to produce spectra of intensity vs uncharged mass (see Supporting Information Section S-3). All single value clusters are deleted from the data structure as these will most likely represent random noise. After charge-state deconvolution, the data is again clustered within the two time domains and the mass domain with *d*_Mah2_ = 0.15. This threshold is larger than the first clustering threshold as the differences within the mass domain are larger than the differences within the *m*/*z* domain and since the single point clusters are removed, there is less chance of false inclusion.

### 3.3 Mass Remainder Strategies

Now that the data structure only consists of clusters that are of interest and their charge states have been reduced, grouping on composition is performed based on MR. Depending on the complexity of a synthetic polymer, *i.e.*, the number of different monomers, the mass of a polymeric unit can generally be expressed as the sum of the mass of the end-groups (α and ω), the mass of the repeating monomers (*m*_rep, *i*_) multiplied by the number of monomers (*n_i_*), and the isotopic variety expressed as the number of carbon-13 atoms multiplied by the difference in mass between ^12^C and ^13^C (*i.e.*, 1.0033 Da), where *i* represents different monomers. Ionization in a mass spectrometer adds the number of charged adducts (*e.g.*, the charge, *z*) multiplied by the adduct mass (*m*_add_) minus the mass of an electron (*m*_e_ = 5.486 × 10^–4^) to the mass and divides the total mass by the charge. For an ion of a homopolymer, this results in eq 1 for the mass-to-charge ratio.
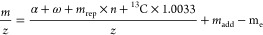
1

**Figure 3:**
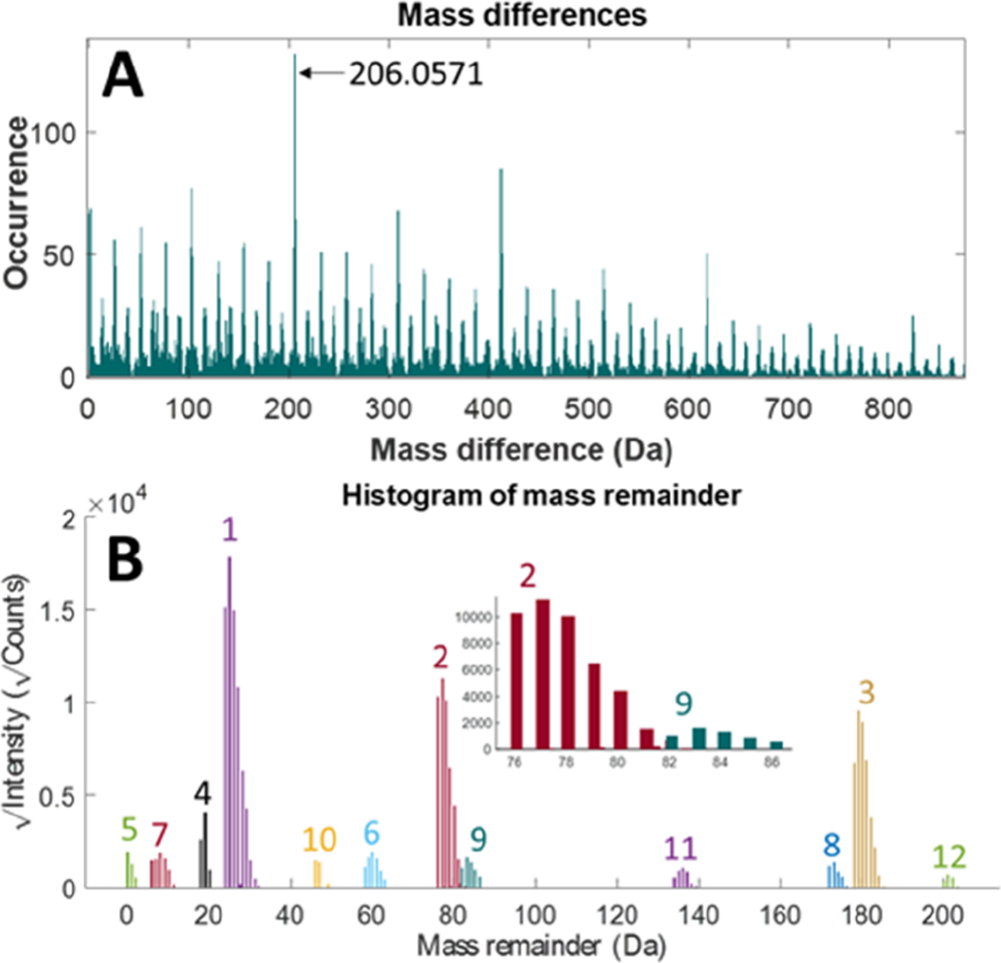
(A) Histogram of mass differences between all found ROIs. The most occurring difference at 206.0571 Da corresponds to the mass of the repeating unit (PG-TPA) of the polyester. (B) Histogram of the found MRs within the polyester data. All groups are numbered from the highest to the lowest intensity. The inset shows a zoomed-in region of the MR plot for series 2 and 9.

The algorithm automatically retrieves the mass of the monomeric units within the polymer by plotting all the mass differences within the ROIs in a histogram. Indeed, together with the mass differences related to isotopic distributions, the monomer mass is the most recurring mass difference in the sample’s spectra. Figure 3A shows the mass-difference histogram of the polyester sample. To avoid the consideration of isotopic contributions (mass differences typically multiples of 1.0033 Da for carbon for instance), the algorithm selects the most abundant mass difference higher than 12 Da. This value was chosen as the smallest monomer unit we may expect in a synthetic organic polymer (*m*_C_ = 12.0000 Da) and may be tuned by the user. The algorithm identified a repeating unit of mass 206.0571 Da. Differences of multiple repeating units (*i.e.*, 2, 3, and 4 repeating units corresponding to 412, 618, and 824 Da) are also found abundantly.

In this work, the sample analyzed is a copolyester produced by the polycondensation of propylene glycol (PG) (*m*(C_3_H_8_O_2_) = 76.0524 Da) and terephthalic acid (TPA) (*m*(C_8_H_6_O_4_) = 166.0266 Da). As a result, the monomers are perfectly alternating and the copolymer can be regarded as a homopolymer with a repeating unit corresponding to the sum of the two monomers minus two water molecules. This value is 206.0579 Da, which is in good agreement (Δ*m* = 8 × 10–4 Da) with the algorithm selection. This functionality is of great interest for analysis where the nature of the polymer is unknown.

The two end-groups of the investigated polyester can consist of any combination of PG, TPA, or HHPA, and the most abundant adduct is sodium (*m*_add_ = 22.9898 Da) in this analysis. After subtracting *m*_add_ – *m*_e_ from each ROI and calculating the MR of each ROI, the resulting MRs were plotted in a histogram (binned with a margin of error of ΔMR = 0.05 Da) against the number of times the MR was found, and results are shown in Figure 3B. This revealed distinct groups each sharing the same end-group composition. Note that the mass of the adduct is an optional user adjustable input parameter and can be set to 0 if multiple or unknown adducts are present. The use of this variable adjusts the mass remainders, so they only contain information about the end-group composition.

### 3.4 Describing the Distributions

Ultimately, we can identify compositional series by grouping the previously found isotope clusters based on their MRs. The decision of whether two isotopic distributions belong to the same compositional series can now be defined by the number of MR values they have in common. The higher chance of incidental overlap for broader isotopic distributions can be compensated for by setting a criterion based on the fraction of MRs that need to overlap instead of their absolute number. Figure S7 in Supporting Information Section S-4 displays the outlined process of grouping the isotopic distributions for a zoomed-in part of the data. The isotopic pattern clusters carry the combined retention and mass-spectral information, though they contain no information about the extent the individual isotopic distributions are related. The MR series yield information on which masses differ by *n* times the repeat unit mass and the end-group compositions; however, MRs are unrelated to the previous chromatographic and mass spectrometric information. Combining the information from both angles effectively groups isotopic distributions to the underlying compositional series 2 and 6. This grouping method is not perfect, especially since the overlap criterion (*C*_ov_) either splits groups into multiple sub-groups with a partial overlap (*C*_ov_ < 0.5) or heavily favors larger isotopic distributions and tends to exclude two-point clusters (*C*_ov_ > 0.5). It can, however, be debated whether two-point isotopic distributions provide enough evidence for identification in the first place. Figure S7D shows the classification of the different compositional series in the polyester data using *C*_ov_ = 0.6. It should be noted that simply comparing the mass remainder of the monoisotopic mass will not result in acceptable group definition as the abundance of the monoisotopic mass decreases quickly with the increasing number of carbon atoms.

### 3.5 Structure Proposal of End-Group Composition

After classification of all compositional series, a structure proposal of these distributions can be performed manually. By cycling through the compositional series, starting with the series with the highest sum intensity, an end-user can form structure proposals for each end-group composition based on the MR. In this step, the MR is expressed as the weighted average of the mono-isotopic mass of the isotopic distribution. One should note that knowledge about the sample is preferable for this step. If libraries of different expected end-group compositions are available, then this step could be performed automatically. The user interface is accompanied with a tool to automatically suggest chemical formulas that are in agreement with the found MR. If the chemical formulas are inconclusive, then the user can decide to add the mass of the repeating unit to the mass (See series 1 and 7 in particular), remove the adduct masses if the initial adduct mass was set to 0, and allow for ion exchange. An example of chemical composition selection can be seen in Supporting Information Section S-5.

**Figure 4:**
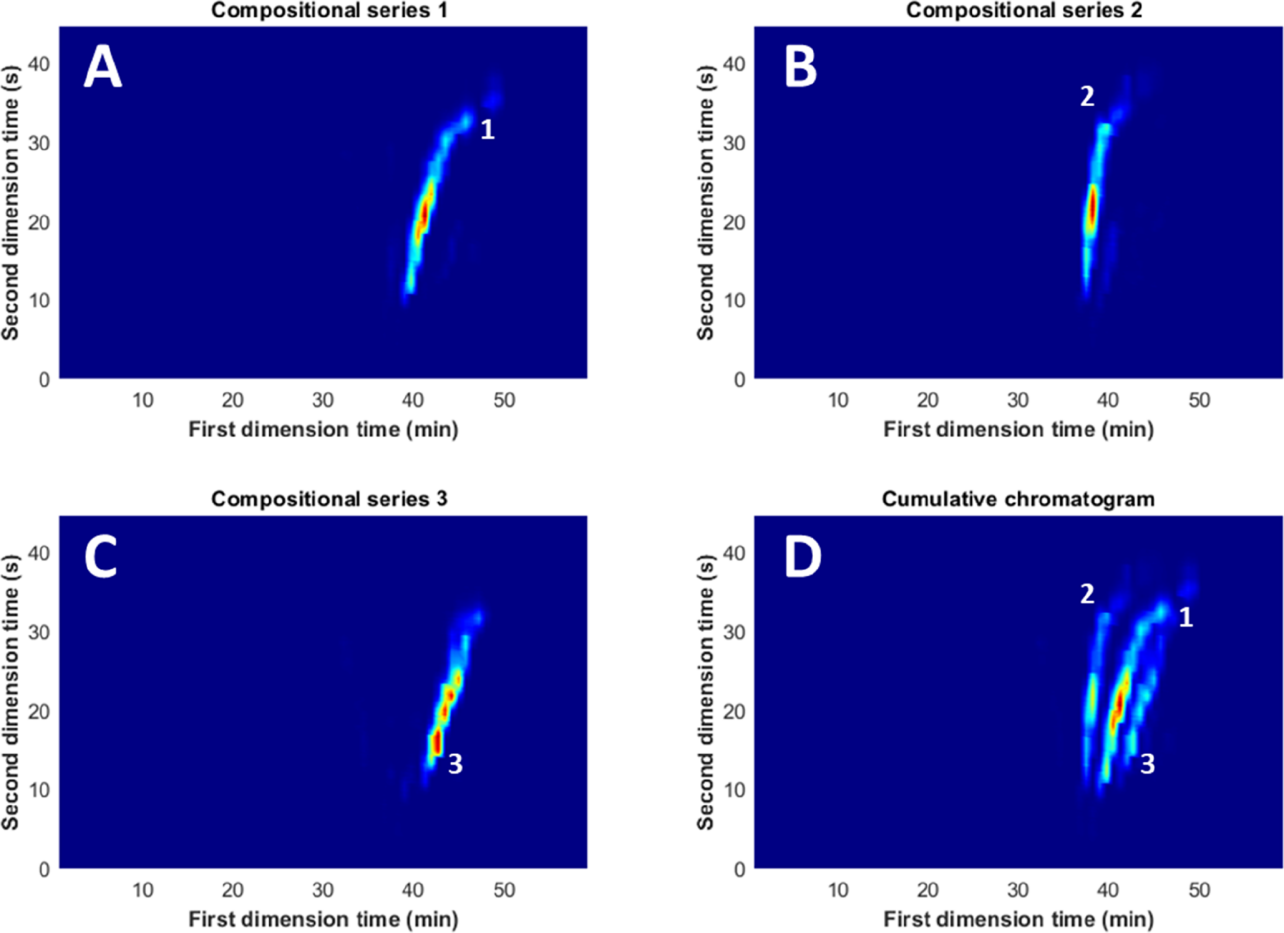
(A) Most prominent compositional series. (B) Second most prominent compositional series. A minor contamination of series 9 is also visible. (C) Third most prominent compositional series. (D) Cumulative chromatogram of the three most prominent compositional series.

**Figure 5:**
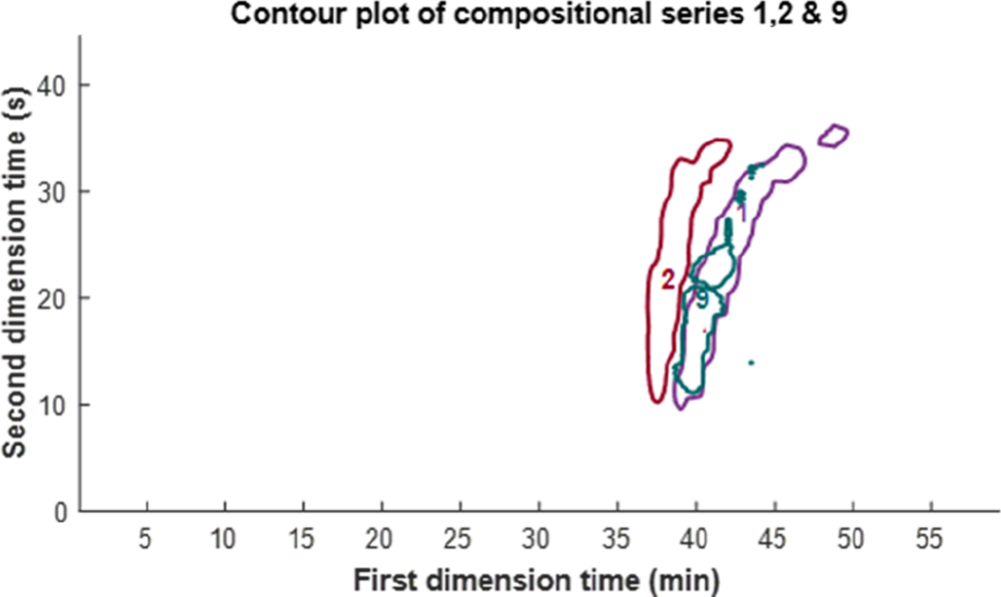
Contour plot of the first, second, and ninth most prominent groups. Group 9 showed chromatographic overlap with group 1 but different chromatographic behavior than the second most prominent group.

Figure 4 shows the three highest sum intensity groups plotted at their positions on the chromatogram and the cumulative chromatogram of these groups. A contour plot of all groups and information about all individual groups can be seen in Supporting Information Section S-5. The algorithm revealed series of polymers that are hard to detect by manual interpretation of the LC × LC–MS data. Figure 5 shows one of these underlying distributions in the form of the ninth most prominent group with an MR of 82.0992 Da. This group co-elutes with the most prominent group and is therefore difficult to distinguish visually within the chromatogram. However, the cumulative MR plot (Figure 3B) clearly shows that this is a different compositional series compared to the chromatographic-related group 1 at MR 24.0685 Da. The distribution of MRs of group 2 starting at 76.0560 Da appears to connect with the distribution of MRs of group 9 (see the inset of Figure 3B); however, it is distinguished as a different group due to the chromatographic behavior (Figure 5). This shows the separation power of the proposed algorithm. With all compositional series mapped, all groups consisting of more than 10 ROIs, where the charge state reduction was successful (*i.e.*, only difference of 1 Da was present within the series, no partial mass differences) and had a unique MR were selected.

**Figure 6:**
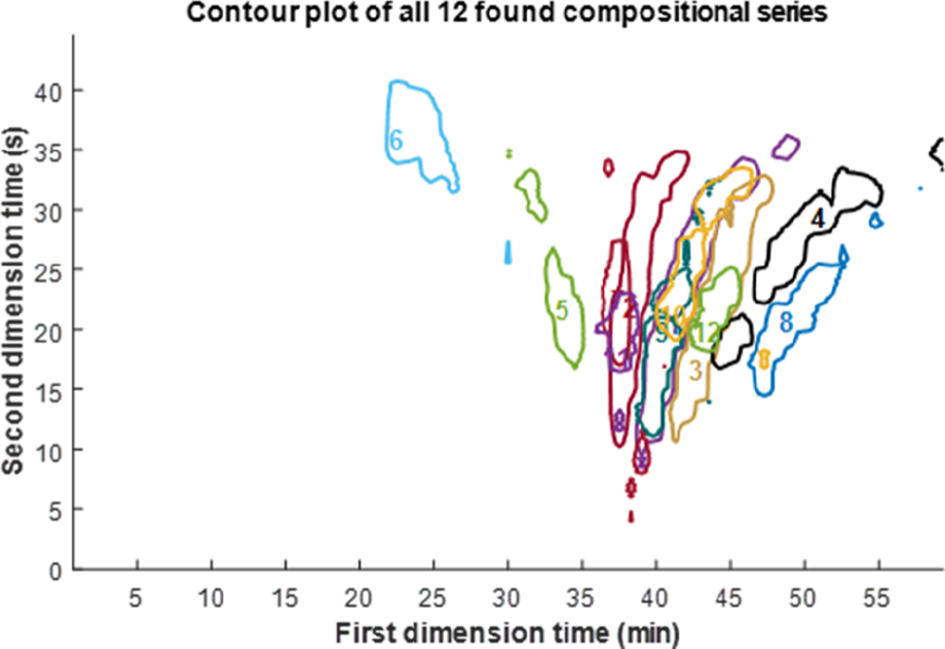
Contour plot of the polyester data showing the approximate positions of the 12 found groups. For a more detailed figure, please refer to the Supporting Information (Figure S8).

In three cases, the algorithm found low abundance distributions with a similar MR and chromatographic location, indicating misclustering. Through the interface that accompanies the algorithm, the user may assess the presence of false positives. The task is made relatively straightforward by a set of plots (see Supporting Information Section S-5). For this case study, this step took approximately 5 min. Figure 6 shows the approximate location in the chromatographic plane of all 12 found groups. Table S2 in Supporting Information Section S-5 shows the MR, proposed end-groups, proposed chemical structures, and mass information for each series.

Since the remaining MR values consist of the sum of both end-group masses, a relatively straightforward interpretation can be done when some background information about the polymer is available. Furthermore, if the chemistry of the sample is not known, or unexpected end-groups are present, then the mass remainder values may provide evidence for possible compositions. The interpretation process for the sample in question was as follows:

The series with an MR of 0.0060 consists only of the repeat unit, and the lack of MR indicates no end-group. In other words, this is a cyclic oligomer. Another series, which behaves chromatographically different and has a different MR of 18.0233 Da, represents a linear polymer consisting of only repeat units and H_2_O (18.0106 Da), that is, PG and TPA are the end-groups (H-(PG-TPA)_*n*_-OH). The MRs of 76.0560 and 172.0762 Da directly correspond to the masses of PG (76.0524 Da) and cyclohexane dicarboxylic acid (172.0736 Da), the reaction product of the derivation with HHPA, indicating dihydroxyl functional end-groups (H-(PG-TPA)_*n*_-PG) and diacid functional end-groups (HHPA-(PG-TPA)_*n*_-OH), respectively. The MR of 24.0685 Da corresponds to an acid/hydroxyl functionality (HHPA-(PG-TPA)_*n*_-PG). This combination is larger than the repeat unit, and thus the resulting MR is aliased; however, it can be calculated that 76.0524 + 172.0736 – 206.0579 – 18.0106 = 24.0575 Da. This conclusion could also be made by realizing that TPA and cyclohexane dicarboxylic acid differ by 6.0470 Da, and thus this difference could be added to the previously determined linear series with an MR of 18.0233 Da. Similarly, the difference of 6.0470 Da can be added to the 172.0762 series to find an HHPA-(PG-TPA)_*n*_-PG-HHPA series with an MR of 178.1253 Da, or this can be concluded by adding two units of HHPA and one unit of PG and extracting two units of water and the repeat unit mass (172.0736 × 2 + 76.0524 – 18.0106 × 2 – 206.0579 = 178.1205 Da). The found series at MRs of 134.1073 and 82.0992 Da show a similar chromatographic behavior to the H-(PG-TPA)_*n*_-PG and HHPA-(PG-TPA)_*n*_-PG series. Compared to these series, they differ by 58.0508 and 58.0307 Da in MR, respectively. Although unexpected, this difference corresponds closely to an extra PG unit in the chain (76.0524 – 18.0106 = 58.0419 Da). This may be caused by a minor contamination of dipropylene glycol in the PG monomer. Dipropylene glycol is a common side product of PG production.^30^

Both MRs of 58.0568 and 6.0557 Da were also found as series and using the same logic as above, these series add an extra PG monomer or replace a TPA unit for an HHPA unit within the cyclic series respectively. The two remaining series, *e.g.*, 46.0489 and 200.1319 Da, show a similar chromatographic behavior to the HHPA-(PG-TPA)_*n*_-PG and HHPA-(PG-TPA)_*n*_-PG-HHPA end-group series. These series differ by 21.9804 and 22.0066 Da in MR, respectively, from their supposedly related group. Sodium exchange, with the free carboxylic acid of the cyclohexane dicarboxylic acid end-group, replaces one of the hydrogen atoms, causing this mass difference with the related group ([R-COOH]Na^+^ + NaX → [R-COONa]Na^+^ + HX) resulting in an exact mass difference of 21.9819 Da (22.9898 Da – 1.0078 Da).

The mass differences between the proposed structures and the experimental data deviate to a varying degree, which can have different explanations. First, the found mass of the repeating unit differs by 0.8 mDa from the theoretical value. Depending on the number of repeating units within the polymeric chain, this difference increases. To accommodate this, the algorithm allows the user to input the true mass remainder if the user deems this necessary. Furthermore, the resolution of the mass spectrometer and the ROI selection can have an impact on the accuracy of the found mass remainders, and especially with low abundant series (*i.e.*, series 12), the ROI selection can be critical in the accuracy of the mass remainders since there are fewer values available to average the MR from.

## 4 Conclusions

An easy and rapid feature mining strategy for LC × LC–MS polymer analysis was successfully developed and applied to an industrial polyester sample. While polymer feature extraction is time-consuming or very often not possible (with traditional MS software), the algorithm classified compositional series within a time span of 3 min, leaving the user with only two tasks: a rapid assessment of false positives (which we performed in 5 min here) and validation of MF assignment. Even series with a low abundancy and high chromatographic overlap with other series were still classified by the algorithm as unique series within the sample. This will allow making better estimations of sample purity and homogeneity within (industrial) samples, which ultimately can provide better tools for product development and quality consistency. Due to the multidimensional technique, varying degrees of information are utilized, *e.g.*, *m*/*z* (and after charge-state reduction, mass), MR, and retention time. This removes the need for deisotoping as the isotopic distributions allow charge-state reduction and simplify the grouping of compositional series, allowing the classification of polymer features.

For all found compositional series that consisted of at least 10 datapoints and where the distribution of MRs consisted of differences of 1 (*i.e.*, successful charge-state reduction), a structure proposal was made. Due to chromatographic overlap, related series with small structural differences (*e.g.*, an additional PG monomer in the chain due to contaminants of dipropylene glycol or sodium exchange within the carboxylic acid groups) were distinguished from each other using the different MRs, whereas compositional series with overlapping MRs were distinguished owing to different chromatographic behaviors. This shows the separation power of the developed algorithm. Confident structural proposals are facilitated by chemical knowledge of the investigated sample; however, on a routine basis, the authors envision setting up custom libraries. Using these libraries, the assignment step can be further supported.

The algorithm, however, is unable to accurately quantify the found distributions. In some cases, the charge-state reduction was incorrect due to small deviations within the *m*/*z* range, leaving some signals unable to be classified within the compositional series. Nonetheless, the information within the selected compositional series can be used to select all *m*/*z* values of interest out of the raw data and perform more accurate quantification of the compositional series.

The algorithm in its current form is viable for homopolymers or perfectly alternating copolymers (*i.e.*, looking at the summed mass of both monomers as the repeating unit). Adaptations of the algorithm are required to accomplish a viable routine analysis for random or block copolymers. Nagy et al. used the MARA technique to distinguish compositional series of copolymers,^12,13,31^ though the end-group composition was not a factor within their sample. When dealing with different chemical compositions and different end-group compositions, additional considerations have to be made since more distributions are present within the sample.

## Supporting Information Available

Visual representation of the feature mining flowchart, background removal, visual representation of charge-state reduction within the data, grouping of information, and information about each found compositional group (PDF)
