# Supplementary material for: Automated Feature Mining for Two-Dimensional Liquid Chromatography Applied to Polymers Enabled by Mass Remainder Analysis
Source: Anal Chem. 2022 Mar 28;94(14):5599–607. doi: 10.1021/acs.analchem.1c05336 (PMC9008690; doi:10.1021/acs.analchem.1c05336)
Supplement: Supplementary file 1 — ac1c05336_si_001.pdf [file ac1c05336_si_001.pdf]

SUPPORTING INFORMATION

Automated feature-mining for two-dimensional liquid chromatography applied to polymers enabled by mass remainder analysis

Stef R.A. Molenaar[1,2,*], Bram van de Put[1,2,3,*], Jessica S. Desport[1,2], Saer Samanipour[1,2], Ron A.H. Peters[1,2,4], Bob W.J. Pirok[1,2]

* Equal contribution
[1] van ‘t Hoff Institute for Molecular Sciences, Analytical Chemistry Group, University of Amsterdam, Science Park 904, 1098 XH Amsterdam, The Netherlands.
[2] Centre for Analytical Sciences Amsterdam (CASA)
[3] TI-COAST, Science Park 904, 1098 XH Amsterdam, The Netherlands
[4] Covestro, Group Innovation, Physics and Material Science, Waalwijk, The Netherlands

Corresponding authors

Stef R.A. Molenaar; e-mail: S.R.A.Molenaar@uva.nl
Bram van de Put; e-mail: Bram.vandePut@wur.nl

Contents

S-1 Visual representation of the feature mining flowchart 3

S-2 Background removal 4

S-3 Visual representation of charge-state reduction within the data..5

S-4 Grouping of information...7

S-5 Information about each found compositional group 8

S-1 Visual representation of the feature mining flowchart

This section illustrates a visual representation of the feature mining algorithm and its user-defined parameters.

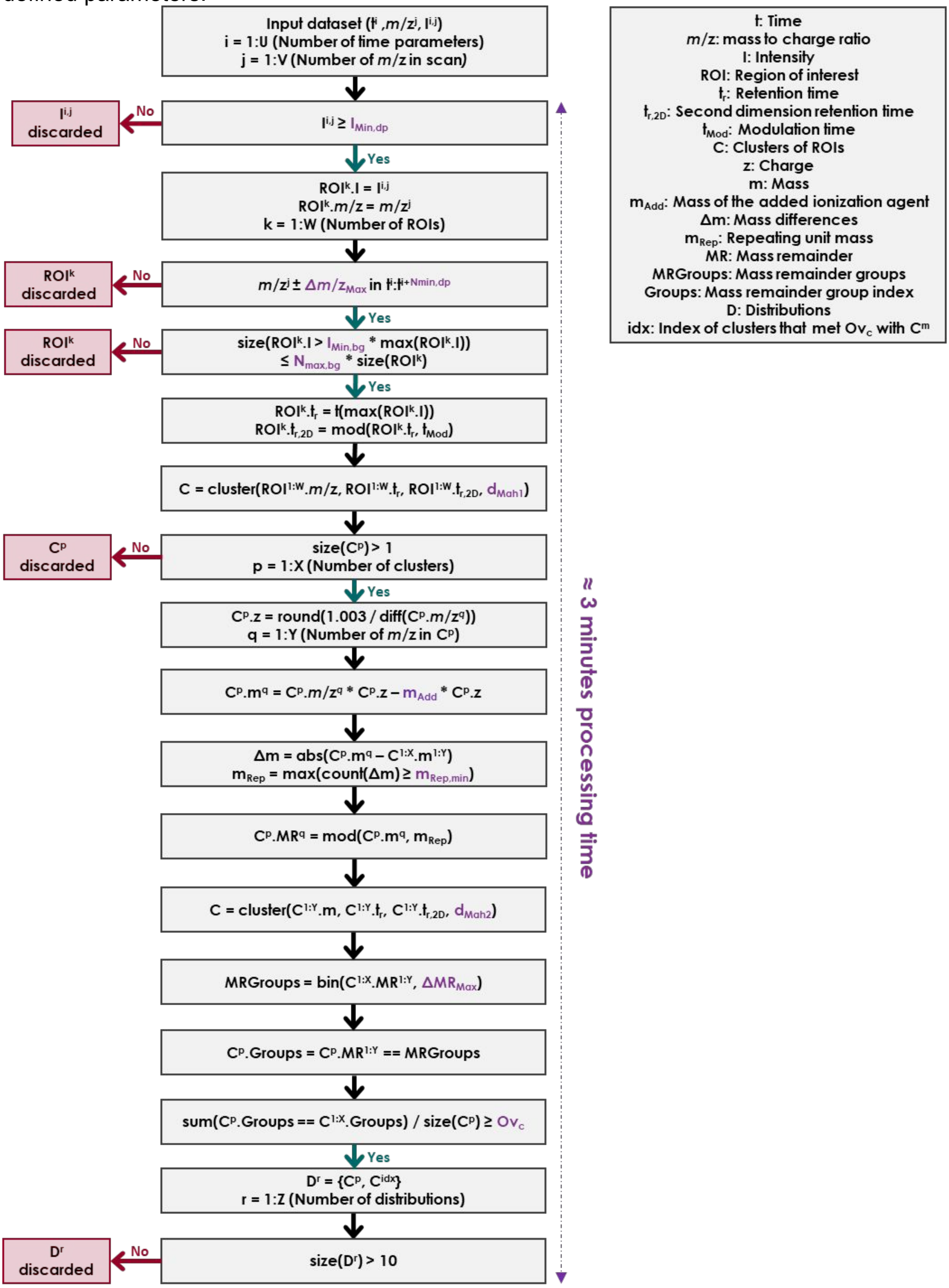

Figure S1. Flowchart for the feature mining algorithm. Purple values are user-defined parameters summarized in table S1 below.

Symbol	Parameter	Value
$I_{Min,dp}$	ROI analysis: Minimum mass peak intensity	100 counts
$\Delta m/z_{Max}$	ROI analysis: Mass tolerance	0.15 Da
$N_{Min,dp}$	ROI analysis: Minimum number of consecutive datapoints	6 scans
$N_{Max,bg}$ || $I_{Min,bg}$	Background removal: Occurrence of signals (*a*) above *b* percent of the maximum ROI intensity	*a* = 2%<br>*b* = 20%
d_{Mah1} || d_{Mah2}	Clustering: Maximum Mahalanobis distance	0.05 || 0.15
$M_{Rep,min}$	MARA: Minimum mass of repeat unit	12.0000 Da
ΔMR_{Max}	MARA: Mass remainder tolerance when binning	0.05 Da
Ov_c	Defining distributions: Overlap criterion	60%
m_{Add}	MARA: Optional parameter. The mass of the adduct	22.9898 Da

Table S1. User-defined parameters used in the algorithm as shown in Fig. S1.

S-2 Background removal

This section provides a visual example of a ROI that was deemed background by the algorithm. In this example 12% of the datapoints within this ROI were higher than the 20% threshold. Meaning this ROI was discarded as background.

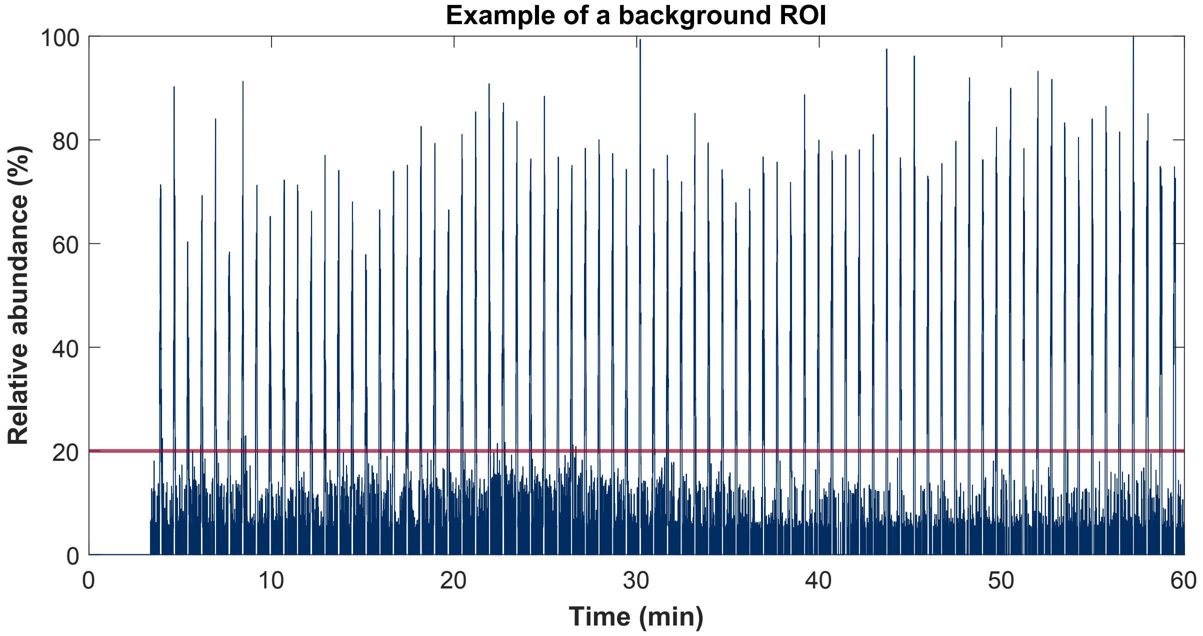

Figure S2. Example of a background ROI. 12% of the intensities of this ROI are higher than the 20% threshold (Red line).

S-3 Visual representation of charge-state reduction within the data

This section illustrates a visual representation of the charge-state reduction within the algorithm. Differences in *m/z* between the isotopic distributions (Fig. S5) can be used to estimate the charge of the measured unit. When multiplying the *m/z* with the charge the mass can be found (Fig. S6)

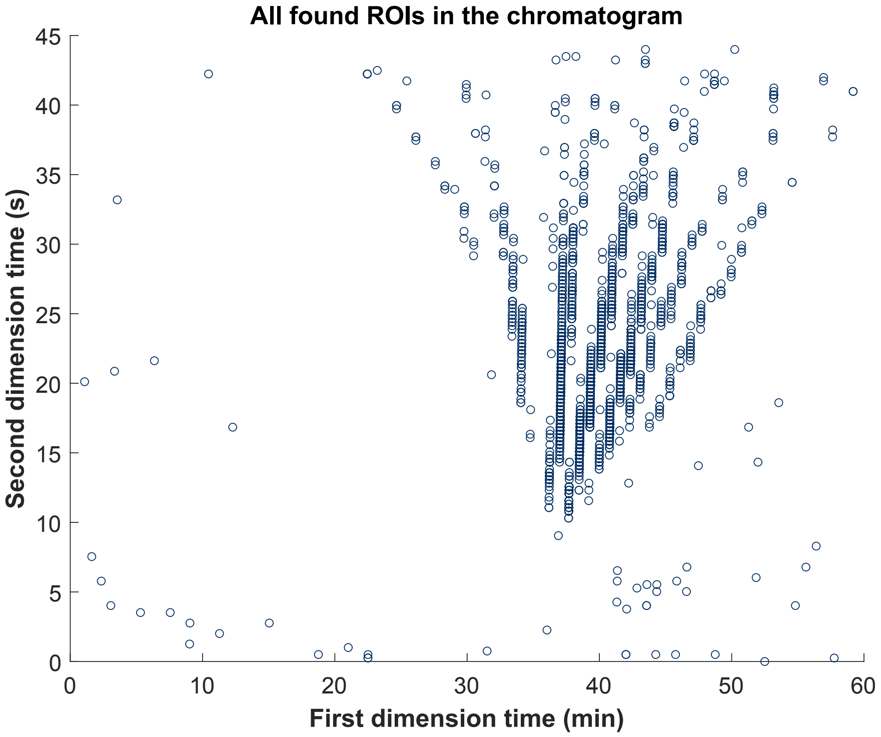

Figure S3: All found ROIs with the LC×LC-MS chromatogram

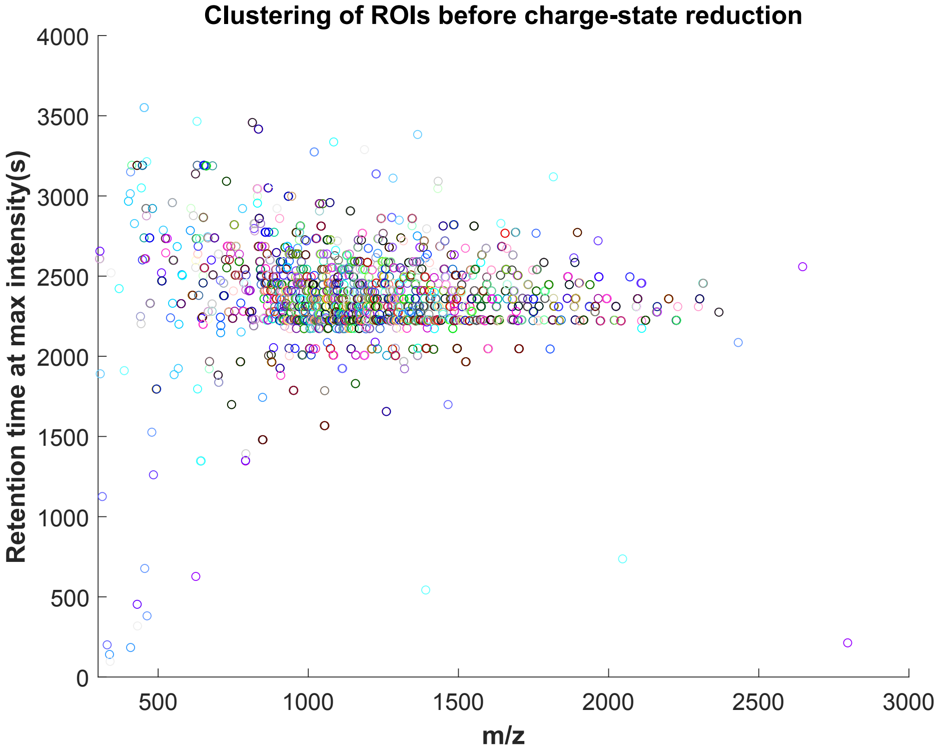

Figure S4: All found ROIs in the time versus m/z domain before processing. Colors indicate grouping by the clustering.

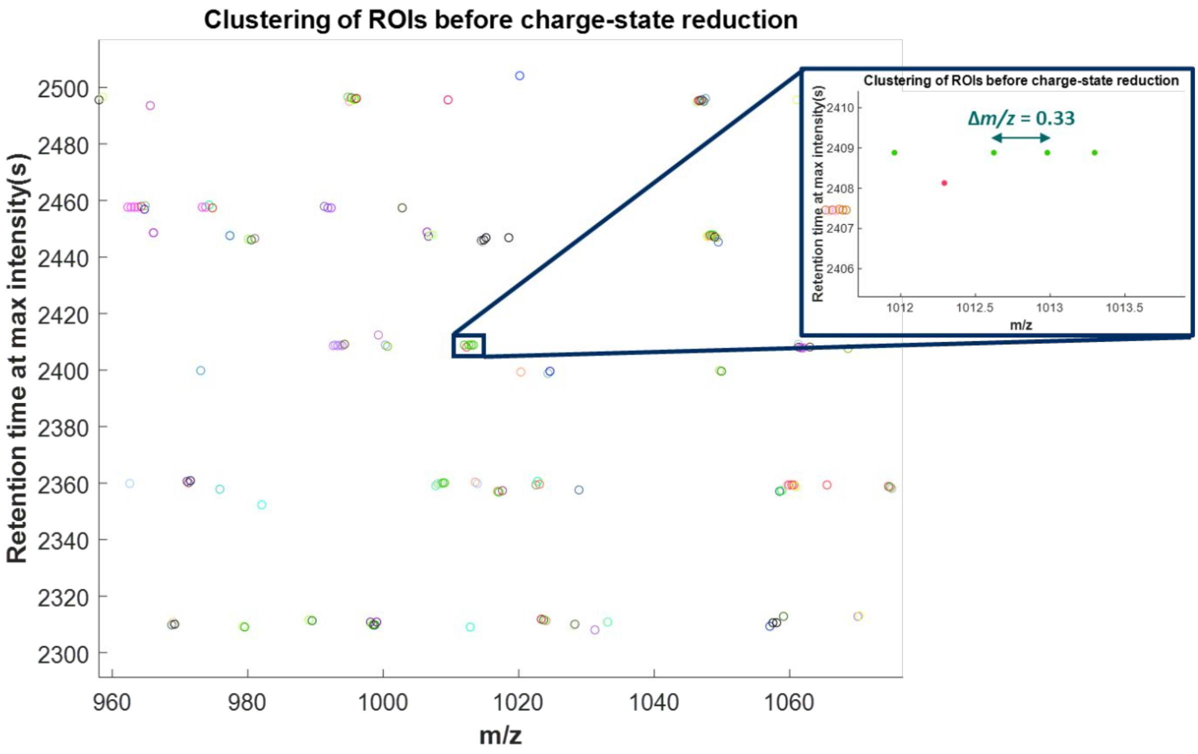

Figure S5: Difference in *m/z* when zoomed in. When the *m/z* difference is equal to $\frac{1}{z}$, the charge state can be found and subsequently the mass of the unit.

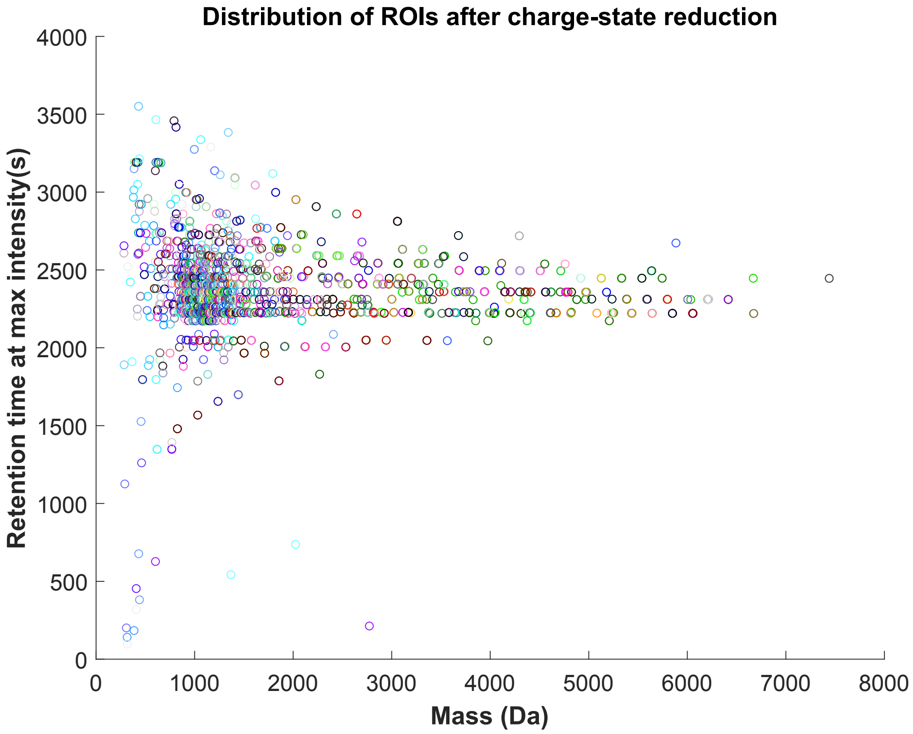

Figure S6: Datapoints in the time versus mass domain after charge-state reduction.

S-4 Grouping of information

This section shows an illustration of grouping of information for series 2 and 6. In figure S7A, the information follows the mass remainder information, whereas in figure S7B the information follows the mass domain. Combining this information leads to the grouping and formation of series 2 and 6 as shown in figure S7C. Figure S7D shows the location of each identified group.

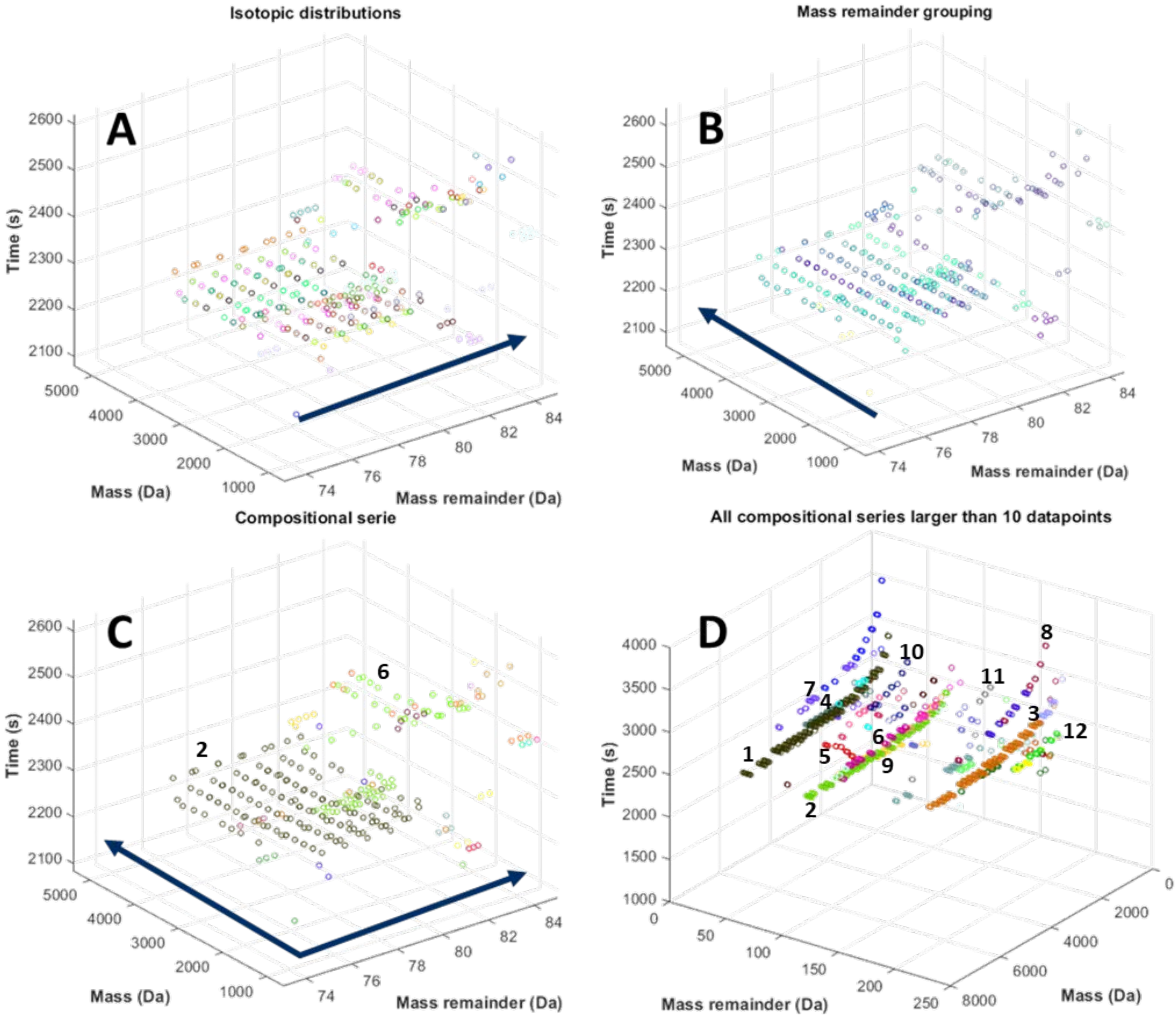

Figure S7. Combining of isotopic and MR information of compositional series 2. Colours indicate different clusters. Arrows point to the direction of grouping in **A**, **B** and **C**. **A**) Grouping by isotopic distribution within a zoomed in regions of the data. Data points with closely related masses are shown as groups follows the mass remainder axis. **B**) Grouping on the MRs. Data points with the same MR are shown as groups follow the mass axis. **C**) Combining the information leads to compositional series 2 in brown within the data. On the top of the figure, a part of compositional series 6 is visible in light green. **D**) Three-dimensional scatter plot of the compositional series within the data structure after combining isotopic and MR information. Positions of each final compositional series is roughly indicated.

S-5 Information about each found compositional group

This section shows information about each compositional group found within the data. Table S2 shows mass remainder and proposed structures and a contour plot showing each selected group and the mass remainder plot are shown in figure S8 and S9. Figure S10 shows the assisted molecular formula determination within the MOREDISTRIBUTIONS user-interface. Figures S11 to S36 show each group and it was selected or not. If it wasn't selected it explains the reason why the group was discarded. Red dots on the chromatogram of the compositional series shows that the group was selected. If the dots remain white, the series was not selected. The red bars on the cumulative mass remainder plot show the newest group of mass remainders. All older mass remainders remain visible as black bars on the plot. Also the native mass spectrum of the group is shown together with the charge-state reduced mass spectrum. Also note that the mass of the ionization unit (M_{Na} = 22.9898 Da) has been removed from this spectrum as well. Most wrongly assessed groups are series wherein the charge-state was not successfully reduced and should have been part of other series. Mass differences between each compositional series should be a list of integers (Or close to 1.0033 Da). When the mass differences plot didn't show good agreement between the found differences (*i.e.* no increments of close to 1), the charge-state deconvolution was deemed wrong. For example, if the mass differences plot showed differences close to 0.5, 1.0, 1.5 and 2.0, a charge of 2 should have been found (As the mass differences increment by ½) and the distribution should have had its charge-state reduced to 1, and thus the found masses should have been doubled. Wrong charge-state reduction most-likely happens due to small deviations within the measured *m/z* values due to the resolution within the mass spectrometer. These small deviations can make it be that the *m/z* difference is not close enough to $\frac{1}{z}$ to be sure about the charge of that particular group. Furthermore, three instances of wrongly assessed groups can be explained due to wrong clustering and the found mass remainder and location on the chromatogram are almost identical to previously determined compositional series and therefore not selected.

No.	Mass remainder (Da)	Proposed end-group	Proposed structure	Δmass (Da) (with related series)	Exact end-group mass (Da)	Mass error (mDa)	M_n (Da)	$Đ_M$	Relative abundance (%)
1	24.0685	HHPA-(PG-TPA)$_n$-PG	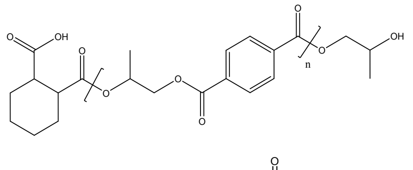	-	24.0575 (230.1154)	11.0	2103	1.35	100
2	76.0560	H-(PG-TPA)$_n$-PG	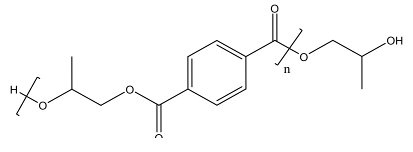	-	76.0524	3.6	1874	1.38	53.4
3	178.1253	HHPA-(PG-TPA)$_n$-PG-HHPA	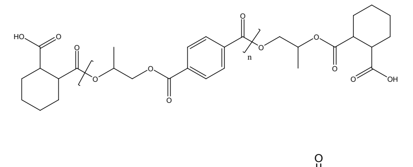	-	178.1205 (384.1784)	4.8	2281	1.20	35.4
4	18.0233	H-(PG-TPA)$_n$-OH	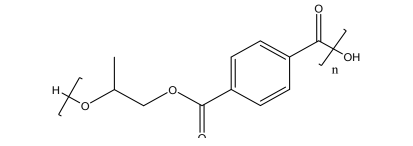	-	18.0106	12.7	1258	1.29	21.2

5	0.0060	No end-group (cyclic)	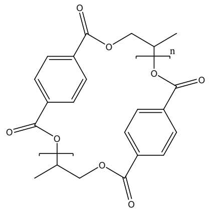	-	0.0000	6.0	709	1.09	5.1
6	58.0568	No end-group (cyclic) + PG	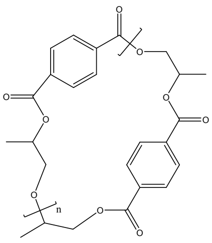	+58.0508 (cyclic)	58.0419	14.9	1825	1.14	4.2
7	6.0557	No end-group (cyclic) + HHPA	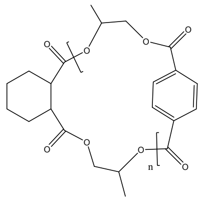	+6.0497 (cyclic)	6.0470 (212.1049)	8.7	1970	1.06	4.2
8	172.0762	HHPA-(PG-TPA)$_n$-OH	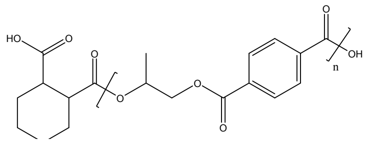	-	172.0736	2.6	2068	1.08	2.6
9	82.0992	HHPA-(PG-TPA)$_n$-PG + PG	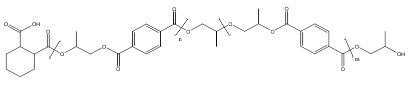	+58.0307 (HHPA-(PG-TPA)$_n$-PG)	82.0994 (288.1573)	-0.2	2693	1.17	2.5
10	46.0489	[HHPA-(PG-TPA)$_n$-PG] Na	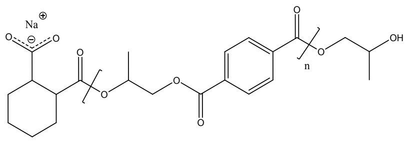	+21.9804 (HHPA-(PG-TPA)$_n$-PG	46.0395 (252.0974)	9.4	1463	1.14	1.9
11	134.1073	H-(PG-TPA)$_n$-PG + PG	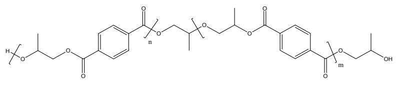	+58.0513 (H-(PG-TPA)$_n$-PG)	134.0943	13.0	2605	1.07	1.4
12	200.1319	[HHPA-(PG-TPA)$_n$-PG-HHPA] Na	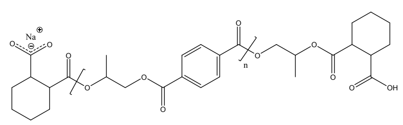	+22.0066 (HHPA-(PG-TPA)$_n$-PG-HHPA)	200.1025 (406.1604)	29.4	2107	1.03	0.6

Table S2: Table of MRs and proposed structures of the 10 compositional series and 2 series that underwent sodium exchange within the mass spectrometer (series 10 and 12) within the copolyester sample. For each series a number, found MR, a proposed end-group composition, a proposed structure, mass difference with related series, the exact mass of the proposed end-groups (with the non-aliased exact mass in parentheses), the mass error, the number average molecular weight, molecular weight dispersity and the relative abundance are given.

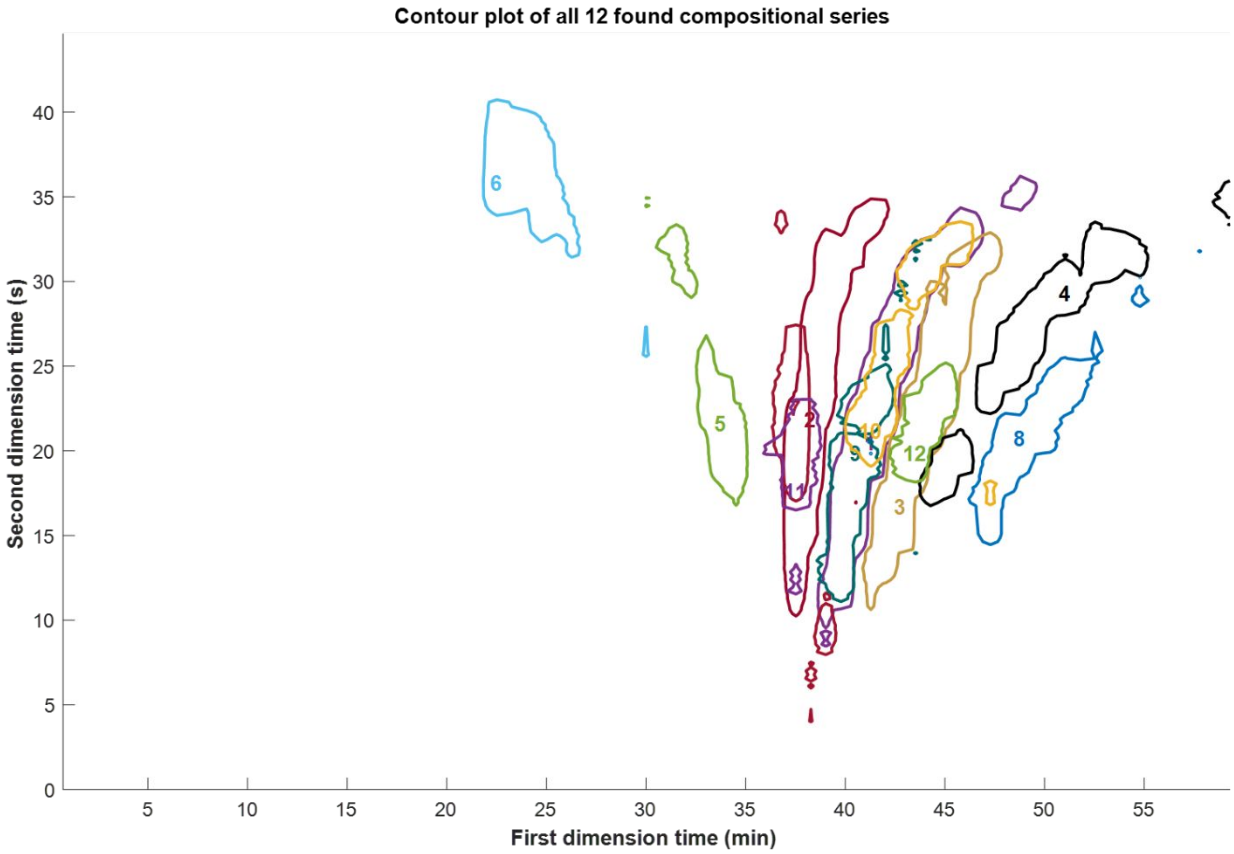

Figure S8: Contour plot of all 12 selected groups. Color and number match with the mass remainder plot as shown in Figure S9.

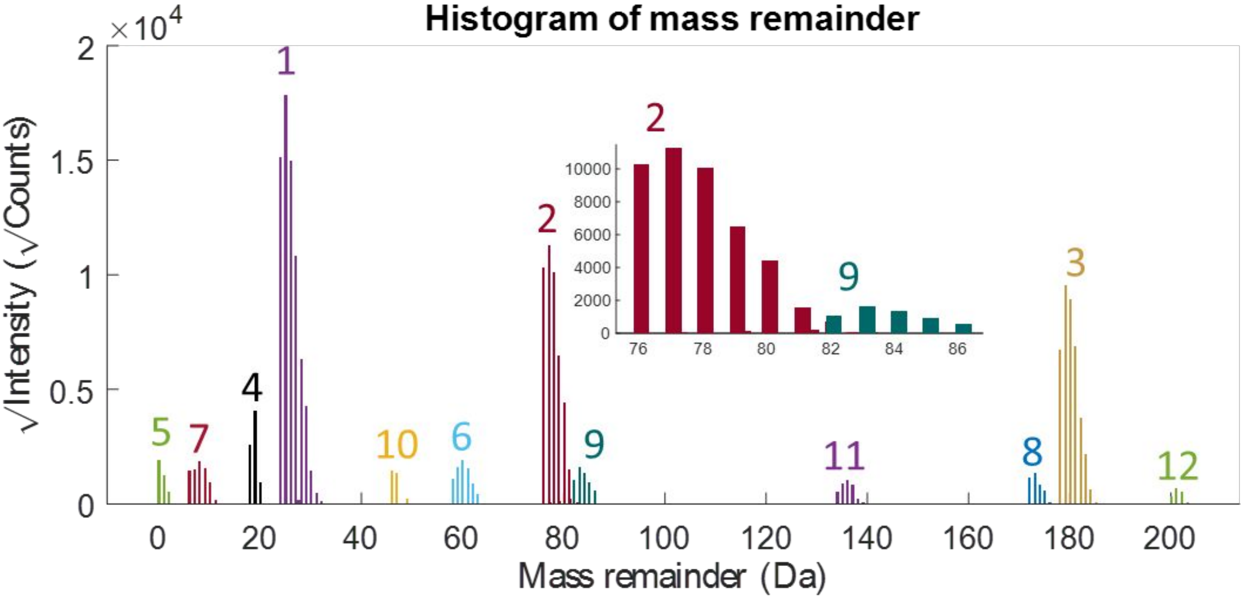

Figure S9: Mass remainder plot of all 12 selected groups. Color and number match with the contour plot as shown in Figure S8.

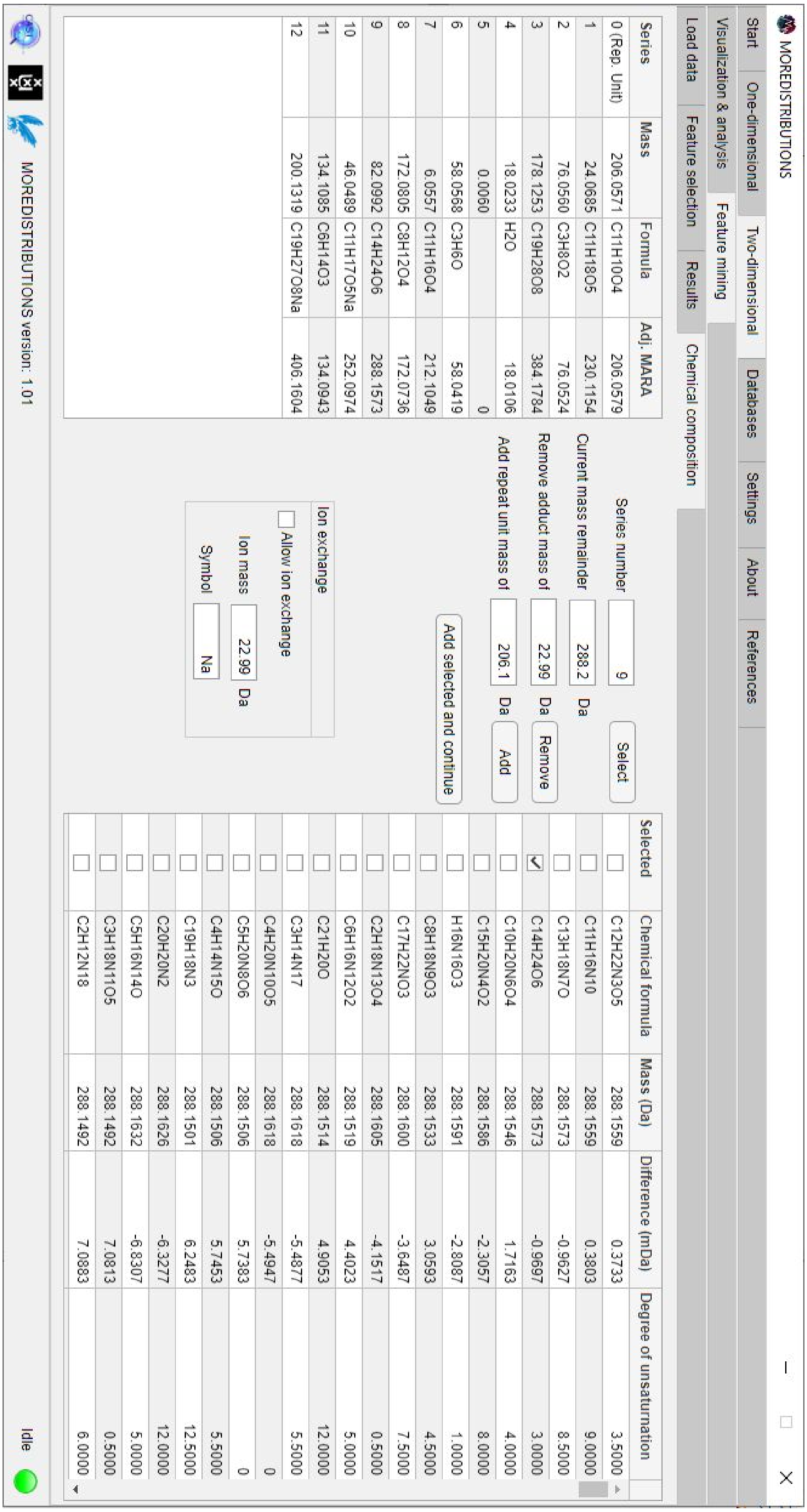

Figure S10: Chemical formula selection within the MOREDISTRIBUTIONS user interface.

Compositional series 1 – Relative abundance: 100% – HHPA-PG

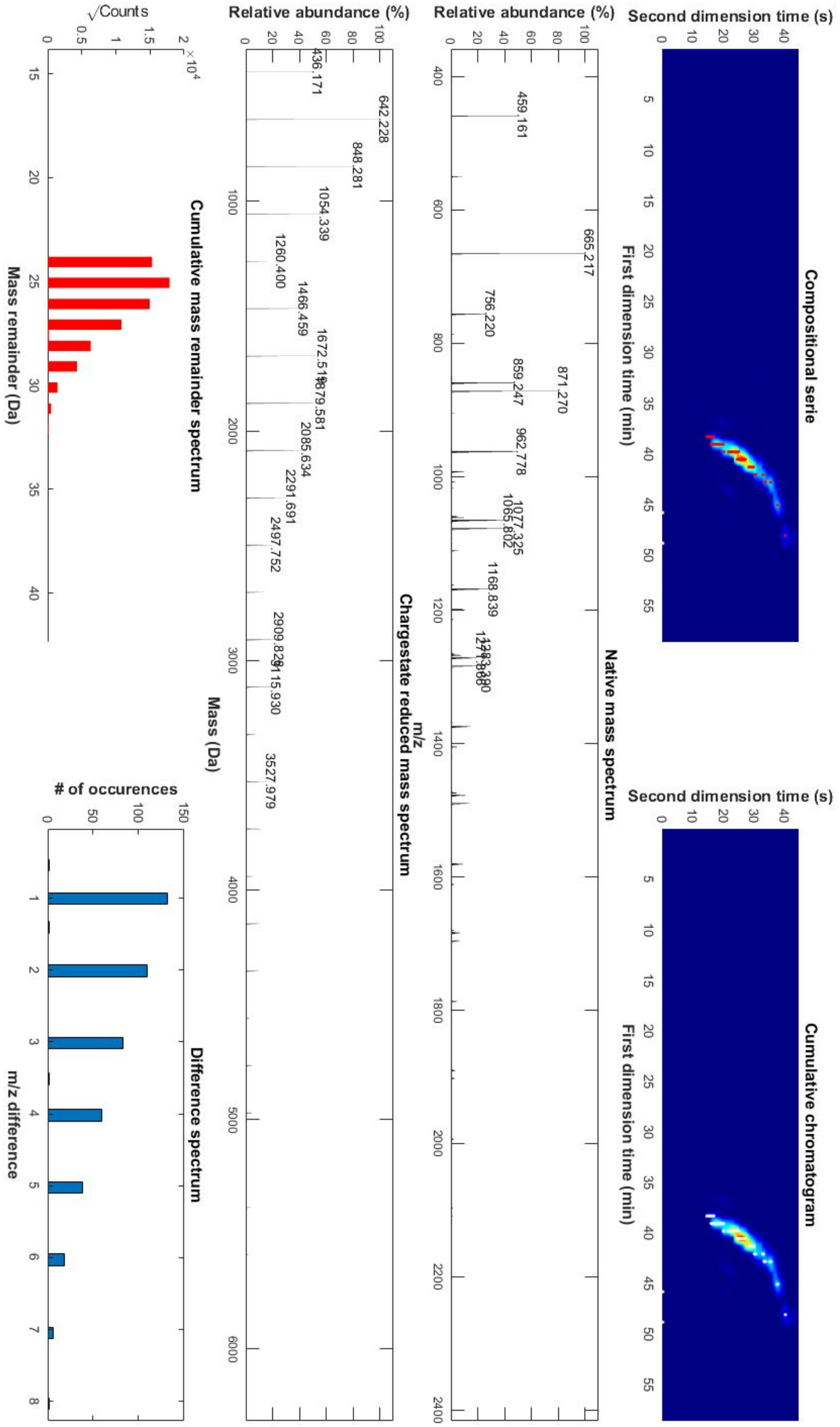

Figure S11: Compositional series number 1. Classified with end-groups HHPA-PG.

Compositional series 2 – Relative abundance: 53.4% – PG-PG

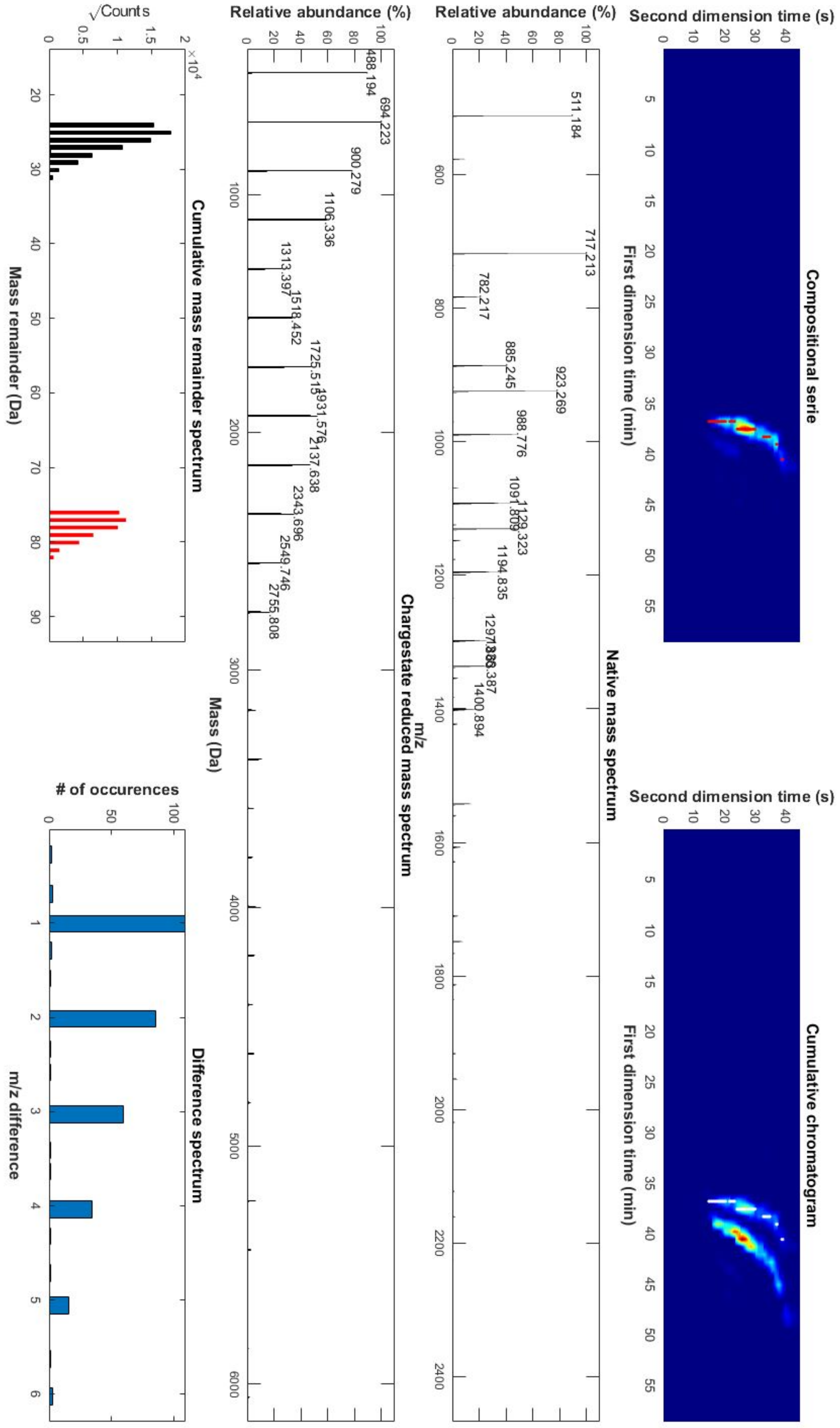

Figure S12: Compositional series number 2. Classified with end-groups PG-PG.

Compositional series 3 – Relative abundance: 35.4% – HHPA-HHPA

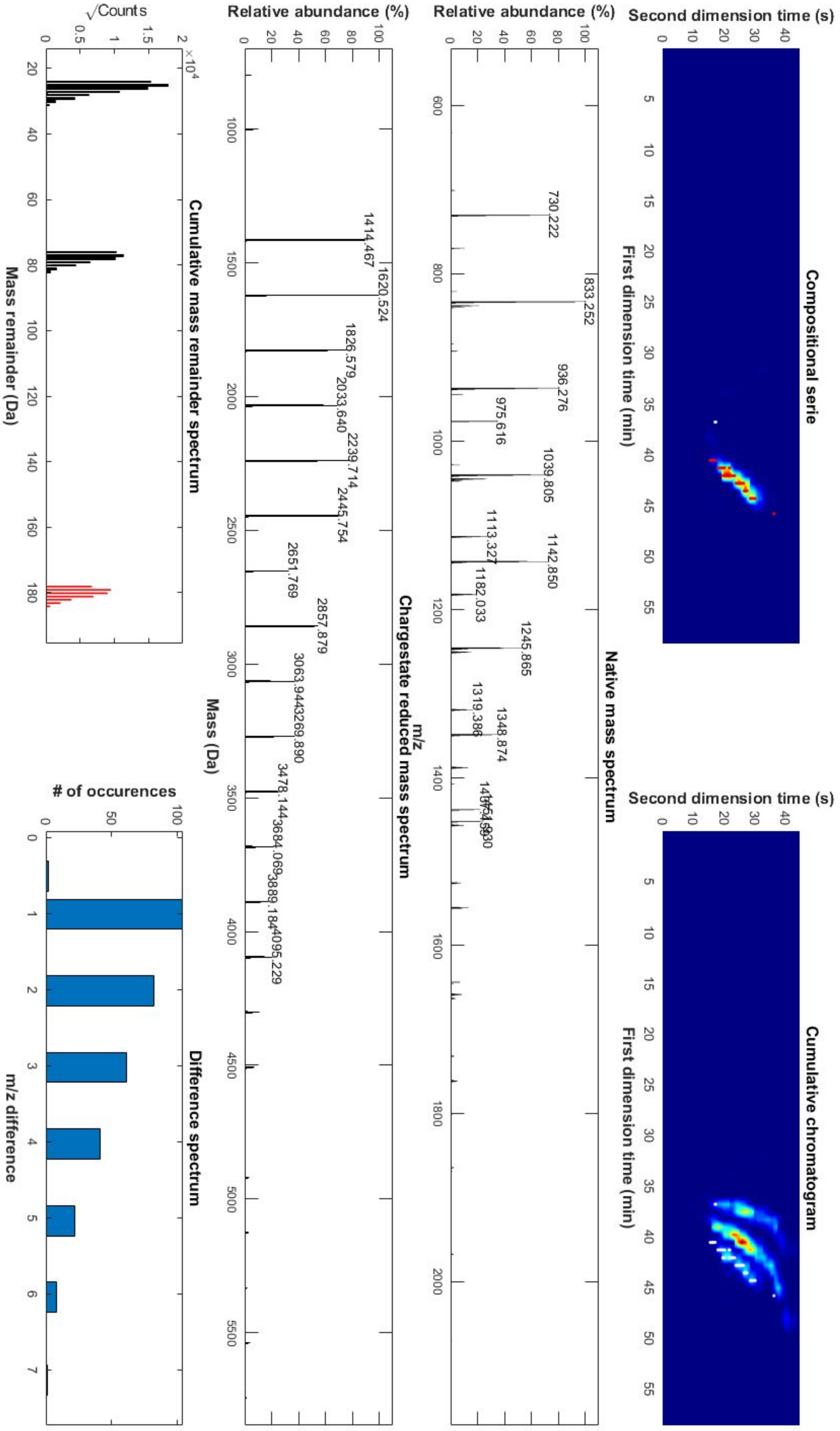

Figure S13: Compositional series number 3. Classified with end-groups HHPA-HHPA.

Compositional series 4 – Relative abundance: 21.2% – TPA-PG (H_2O)

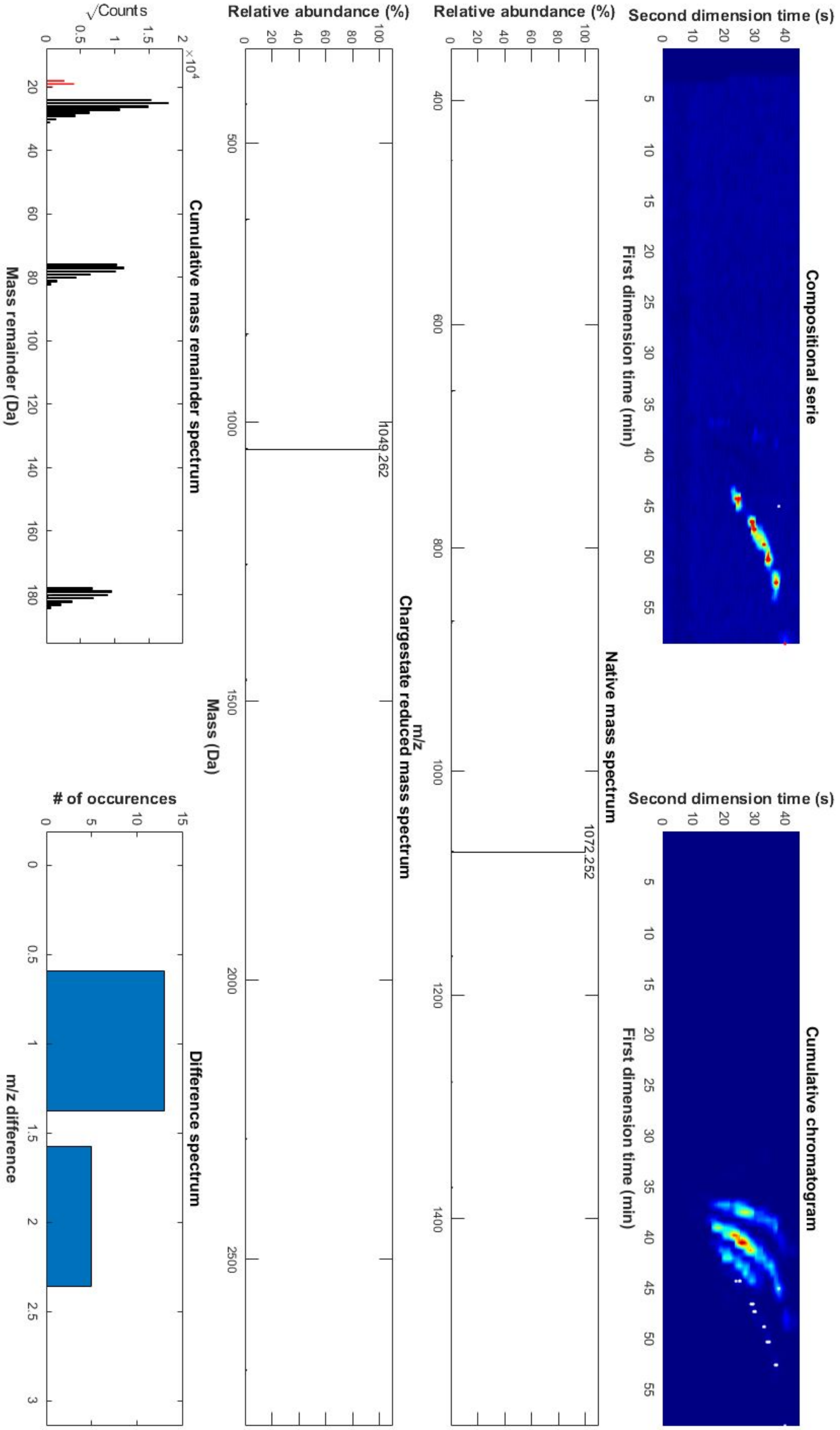

Figure S14: Compositional series number 4. Classified with end-groups TPA-PG, or H_2O.

Wrongly assessed series 1 – Relative abundance: 10.3% – Mass remainder already found

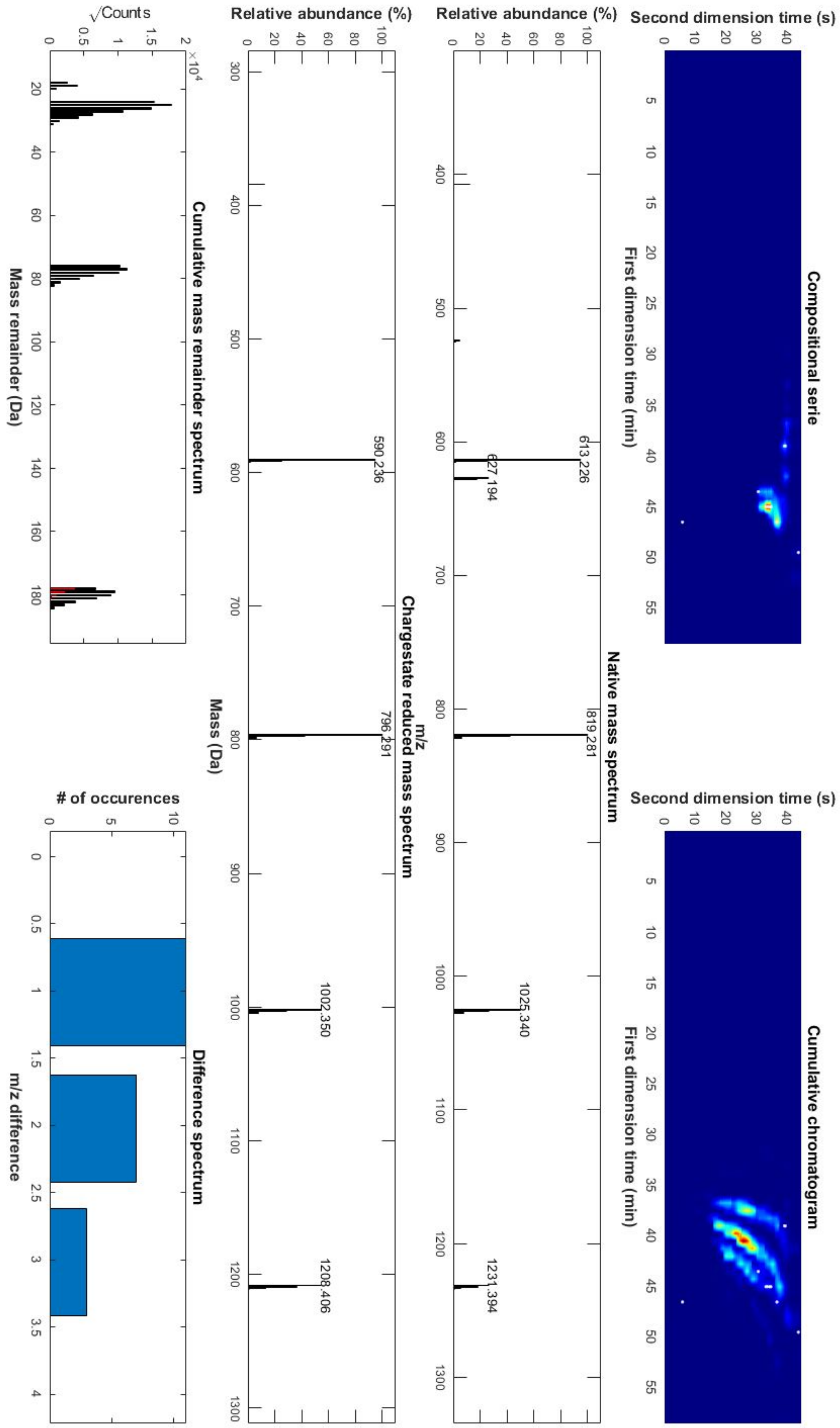

Figure S15: Wrongly assessed compositional series number 1. Mass remainder was identical and strong chromatographic has overlap with compositional series 3 (Fig. S13).

Compositional series 5 – Relative abundance: 5.1% – No end-group (Cyclic)

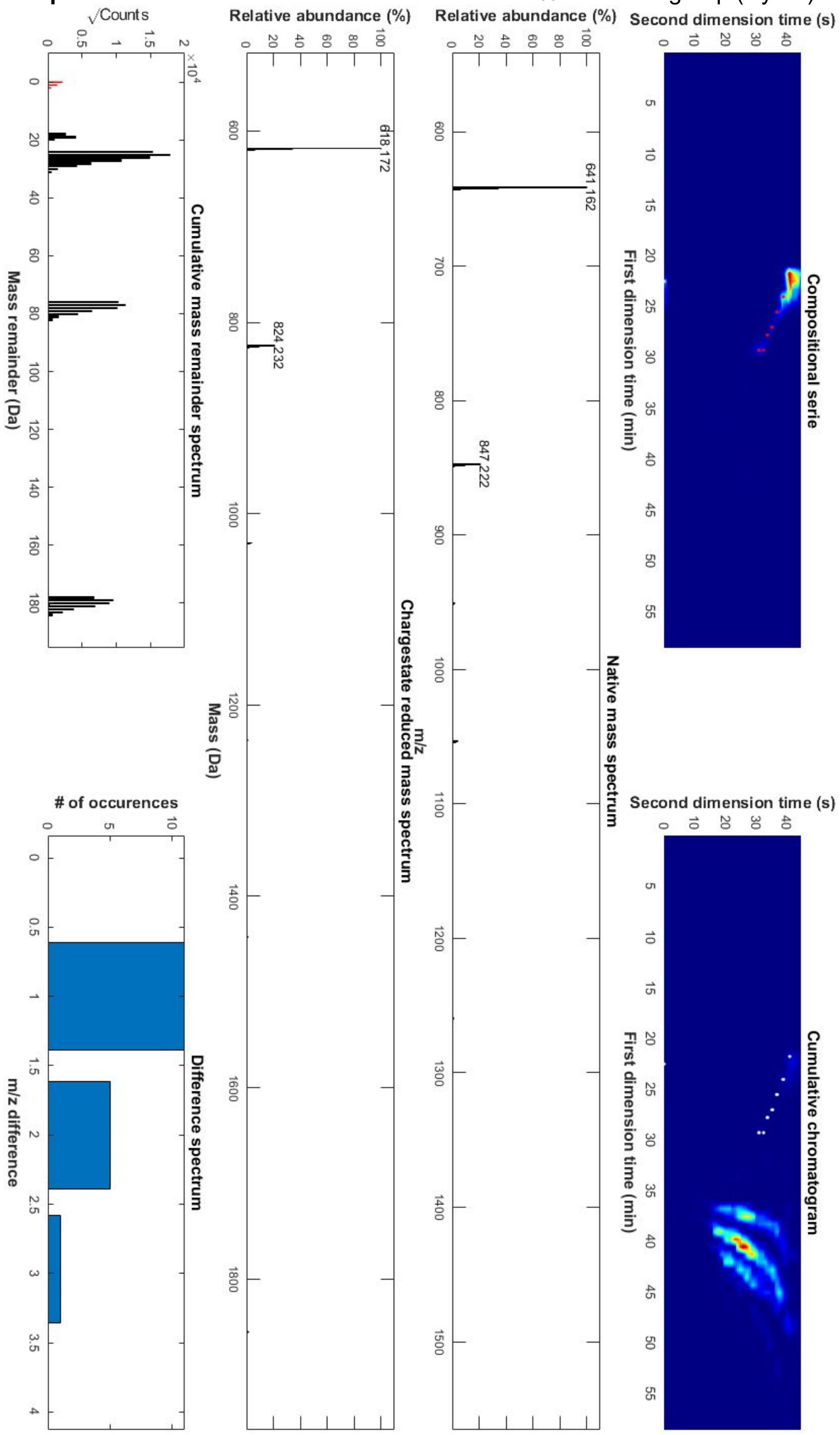

Figure S16: Compositional series number 5. Classified with no end-groups, or cyclic.

Wrongly assessed series 2 – Relative abundance: 4.3% – Incorrect charge-state reduction

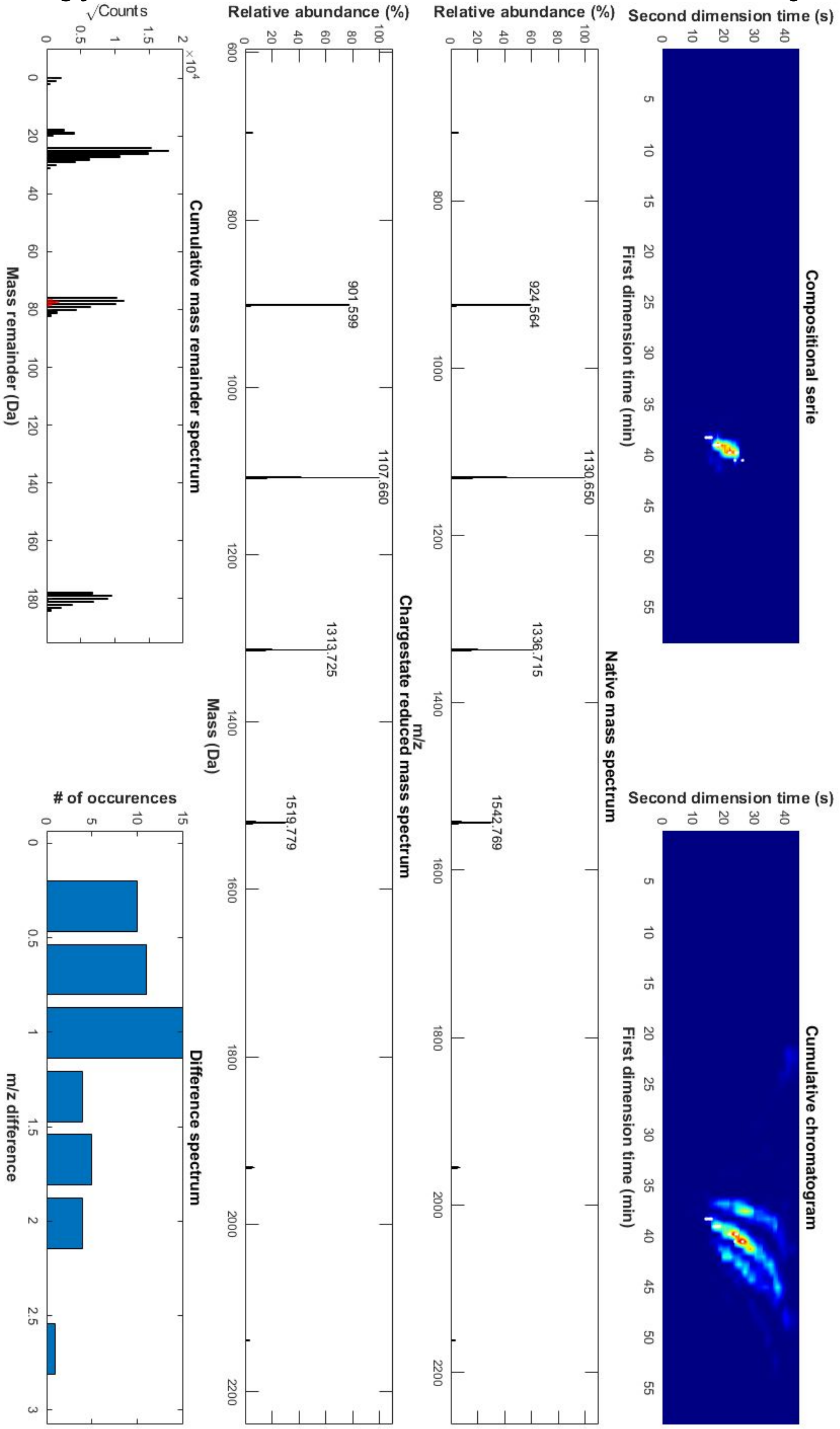

Figure S17: Wrongly assessed compositional series number 2. Charge-state reduction was poorly performed, additionally the mass remainder was already found.

Compositional series 6 – Relative abundance: 4.2% – No end-group (Cyclic) + PG

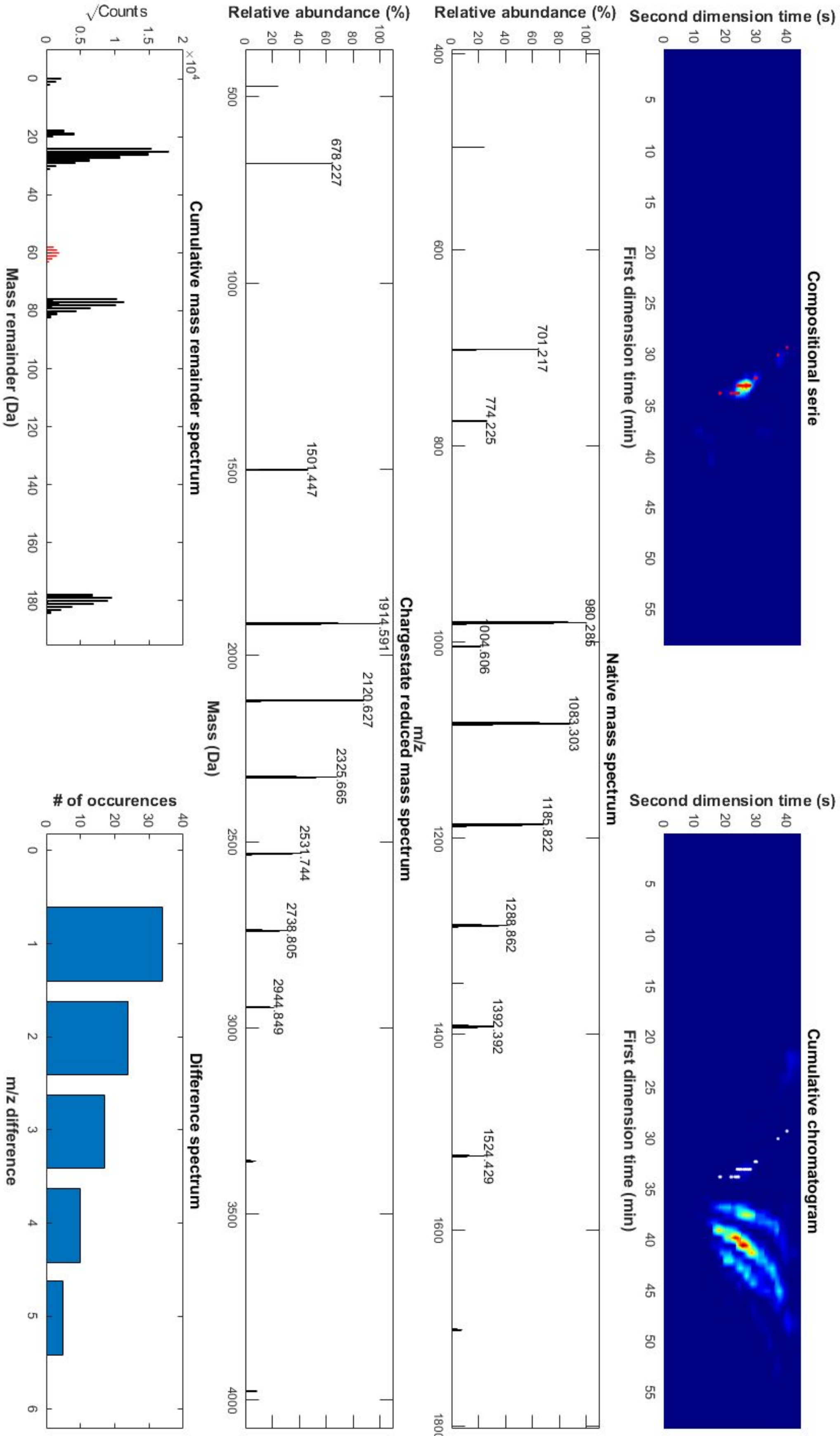

Figure S18: Compositional series number 6. Classified with no end-groups, or cyclic, but with an additional PG group within the polymeric chain.

Compositional series 7 – Relative abundance: 4.2% – No end-group (Cyclic) + HHPA

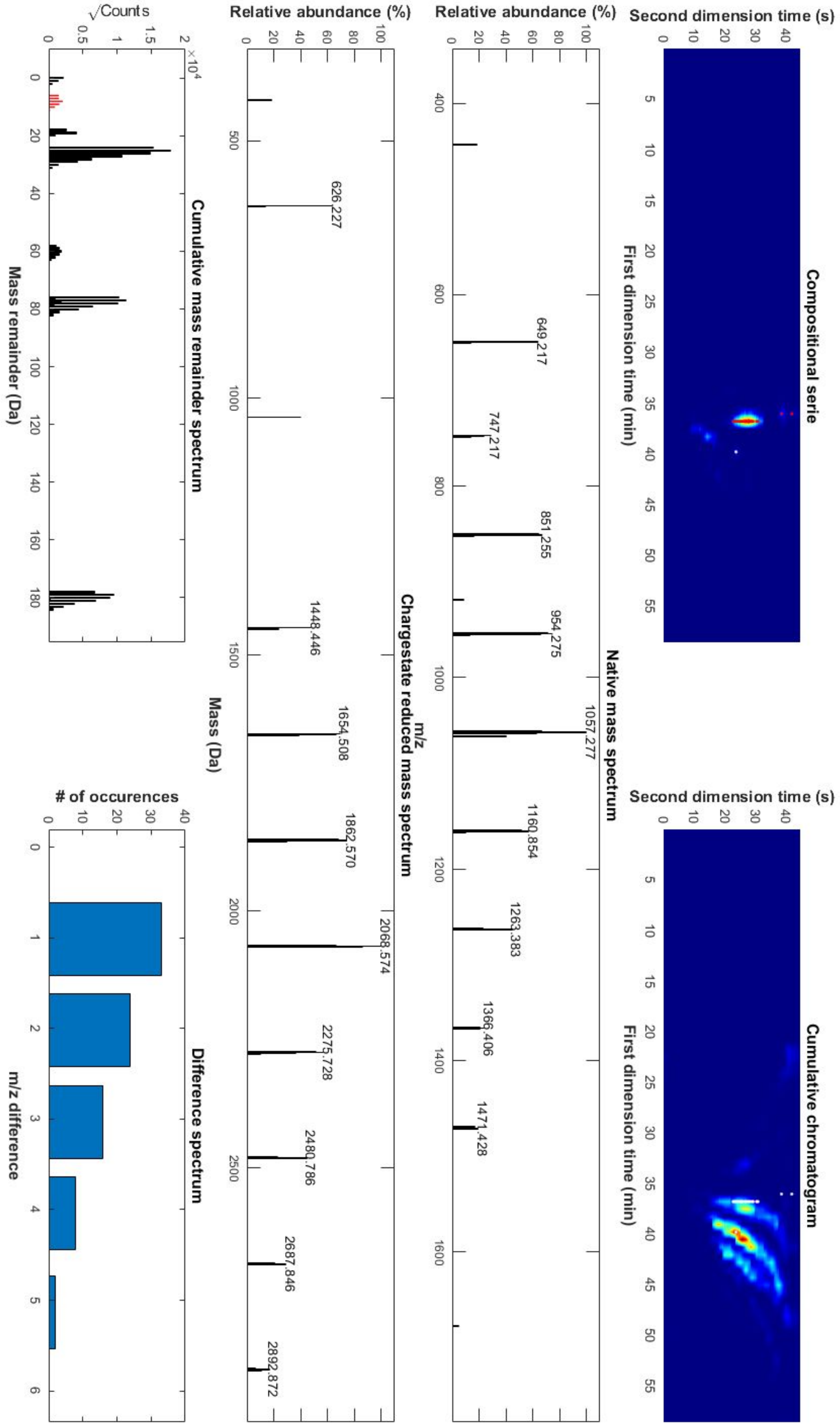

Figure S19: Compositional series number 7. Classified with no end-groups, or cyclic, but with an HHPA group within the polymeric chain.

Wrongly assessed series 3 – Relative abundance: 3.1% – Mass remainder already found

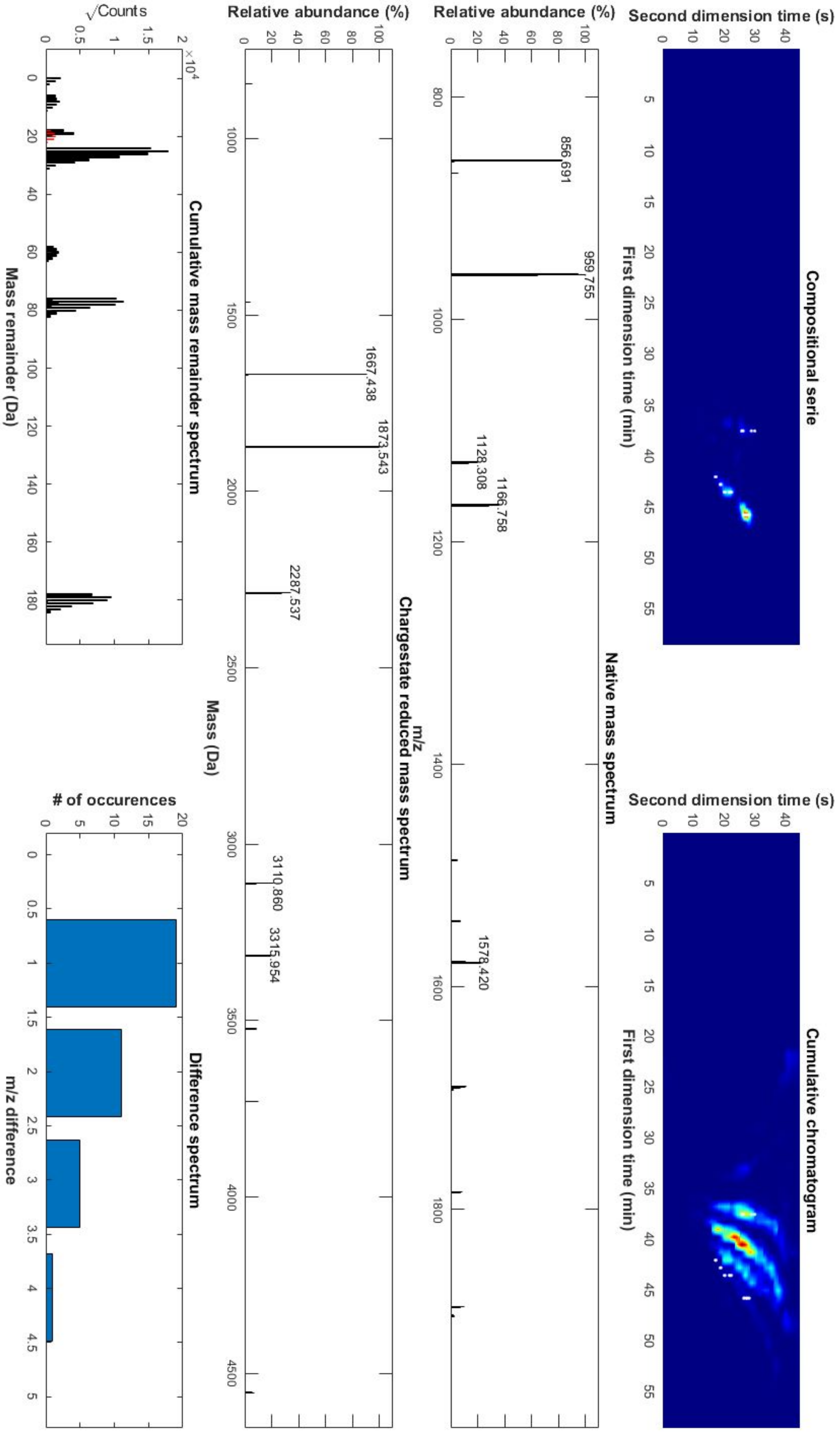

Figure S20: Wrongly assessed compositional series number 3. Mass remainder was identical and strong chromatographic has overlap with compositional series 4 (Fig. S14).

Compositional series 8 – Relative abundance: 2.6% – HHPA-TPA

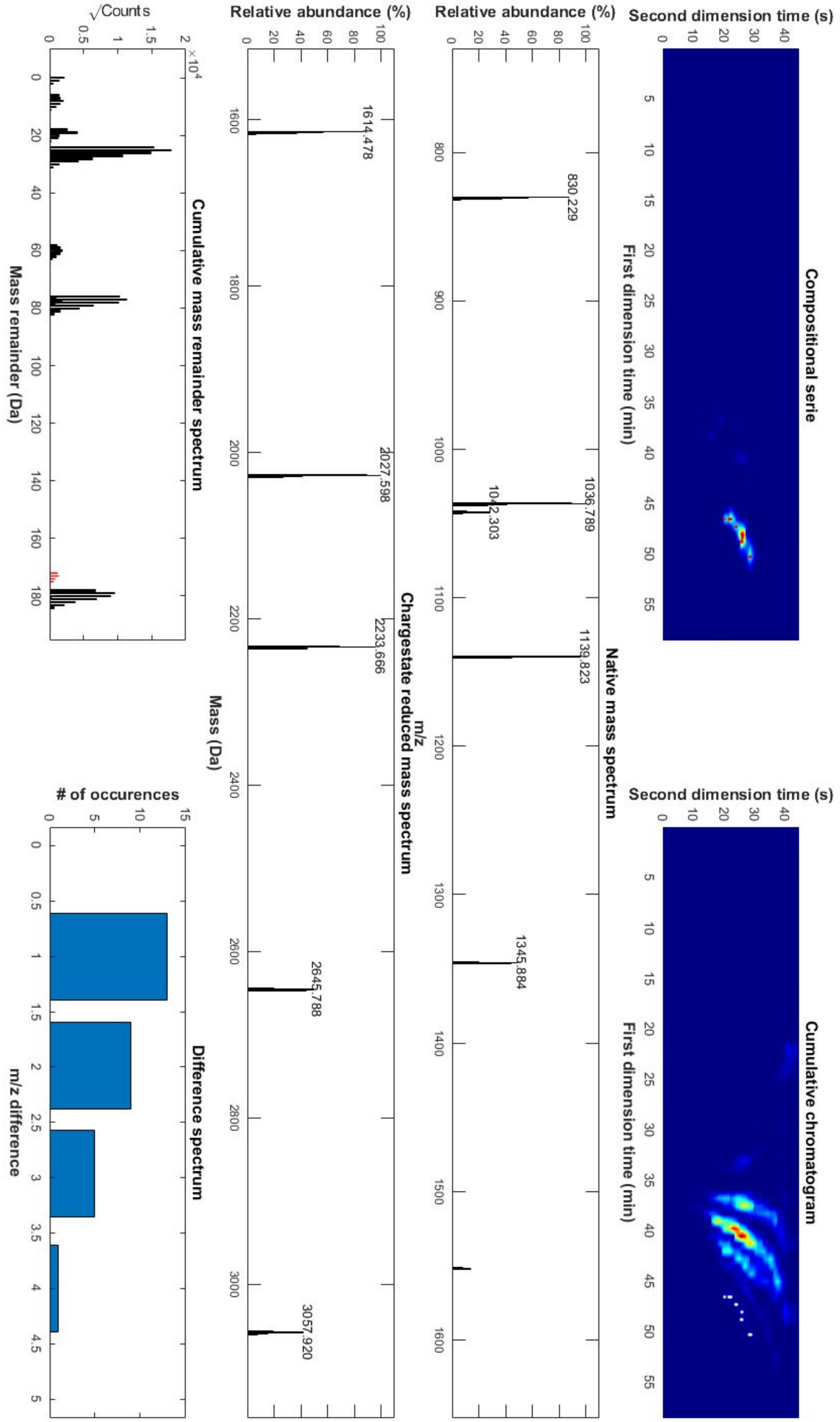

Figure S21: Compositional series number 8. Classified with end-groups HHPA-TPA.

Compositional series 9 – Relative abundance: 2.5% – HHPA-PG + PG

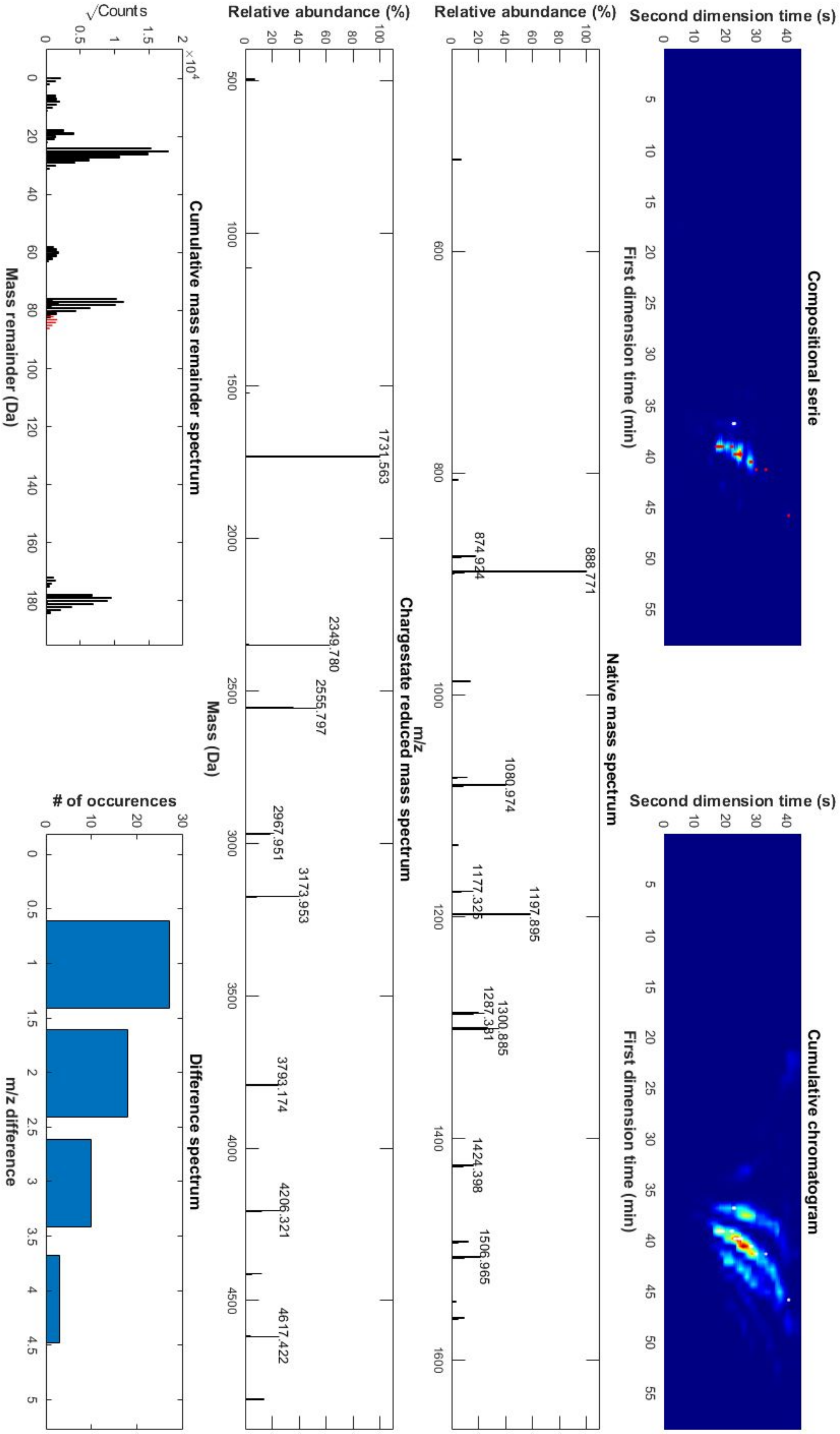

Figure S22: Compositional series number 9. Classified with end-groups HHPA-PG, but with an additional PG group within the polymeric chain.

Wrongly assessed series 4 – Relative abundance: 2.0% –Incorrect charge-state reduction

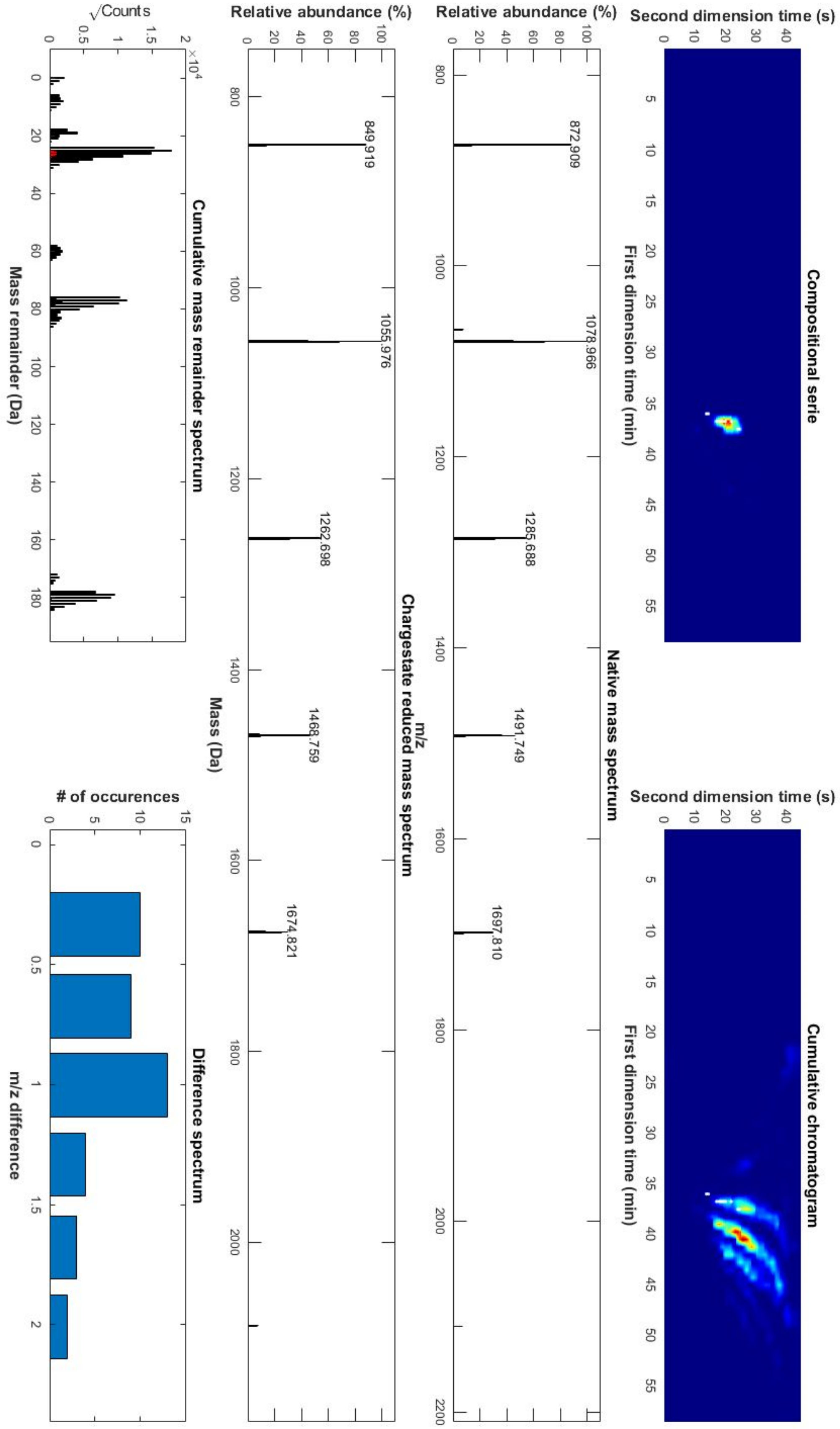

Figure S23: Wrongly assessed compositional series number 4. Charge-state reduction was poorly performed, additionally it has strong chromatographic overlap with compositional series 1 (Fig. S11) with the same mass remainder.

Wrongly assessed series 5 – Relative abundance: 2.0% –Mass remainder already found

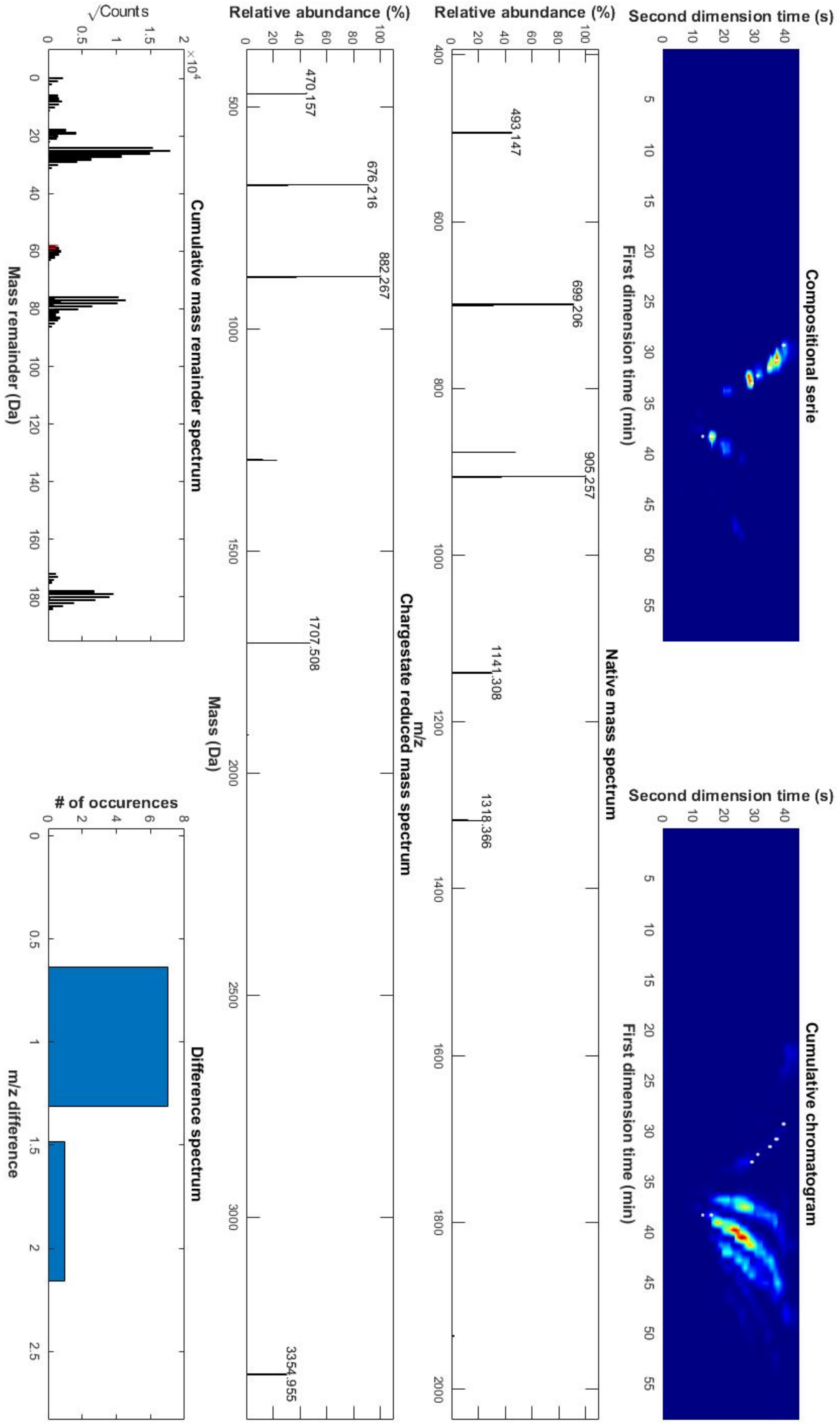

Figure S24: Wrongly assessed compositional series number 5. Mass remainder was identical and strong chromatographic has overlap with compositional series 6 (Fig. S18).

Compositional series 10 – Relative abundance: 1.9% – [HHPA-PG]Na

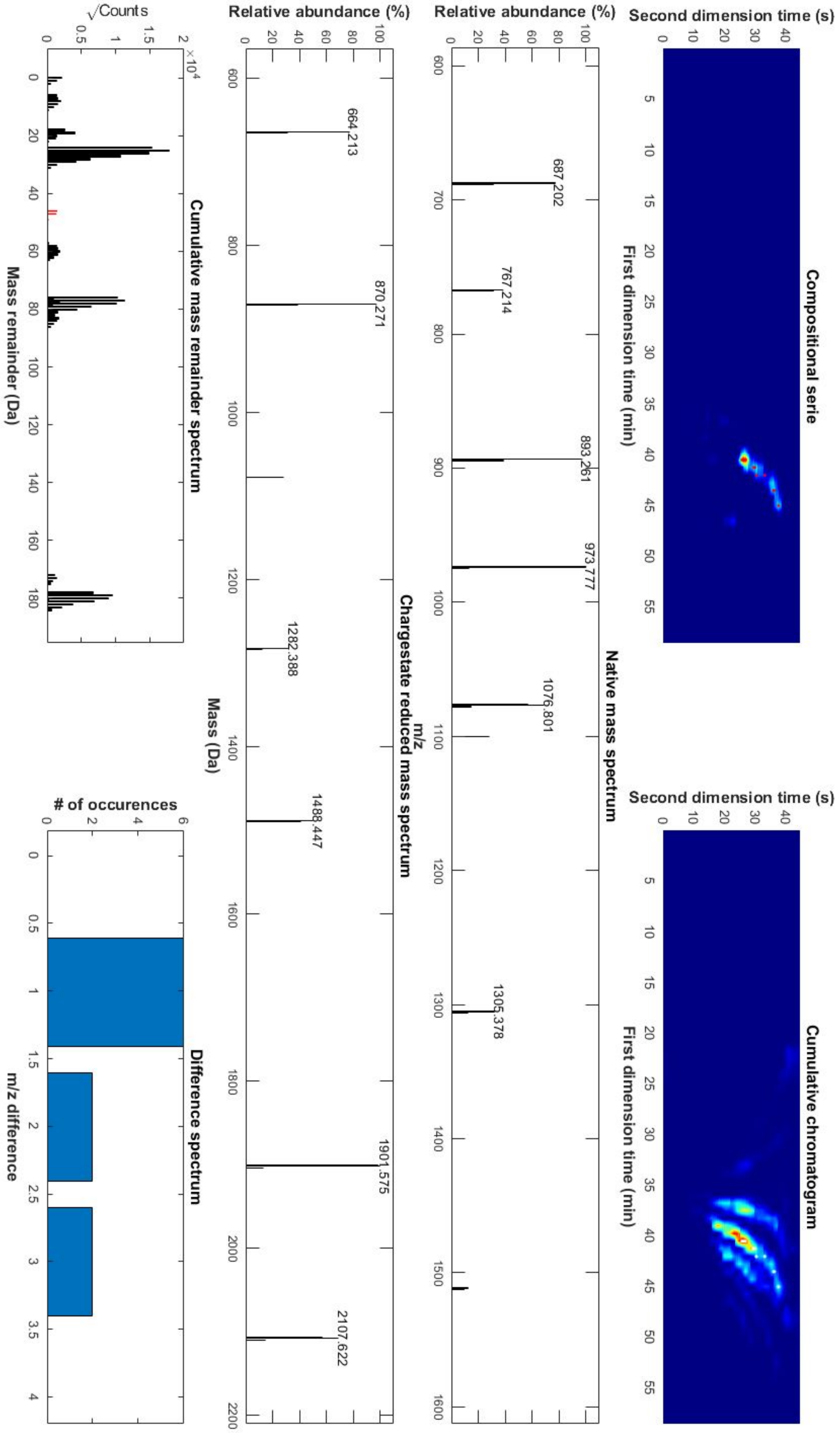

Figure S25: Compositional series number 10. Classified with end-groups HHPA-PG, but underwent sodium exchange within the free carboxylic acid of the cyclohexane dicarboxylic acid (Reaction product of the derivation with HHPA).

Wrongly assessed series 6 – Relative abundance: 1.9% –Incorrect charge-state reduction

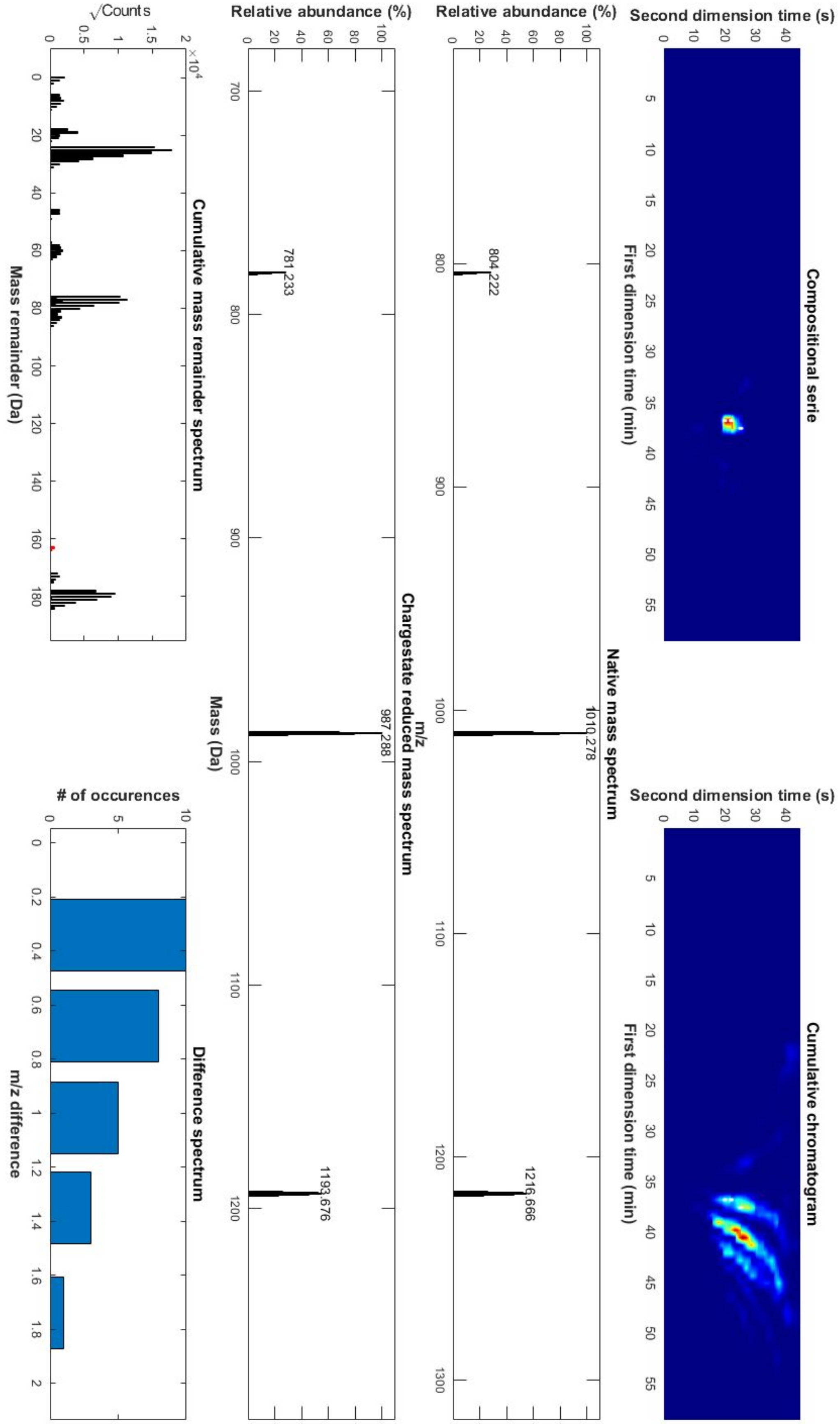

Figure S26: Wrongly assessed compositional series number 6. Charge-state reduction was poorly performed.

Wrongly assessed series 7 – Relative abundance: 1.6% –Incorrect charge-state reduction

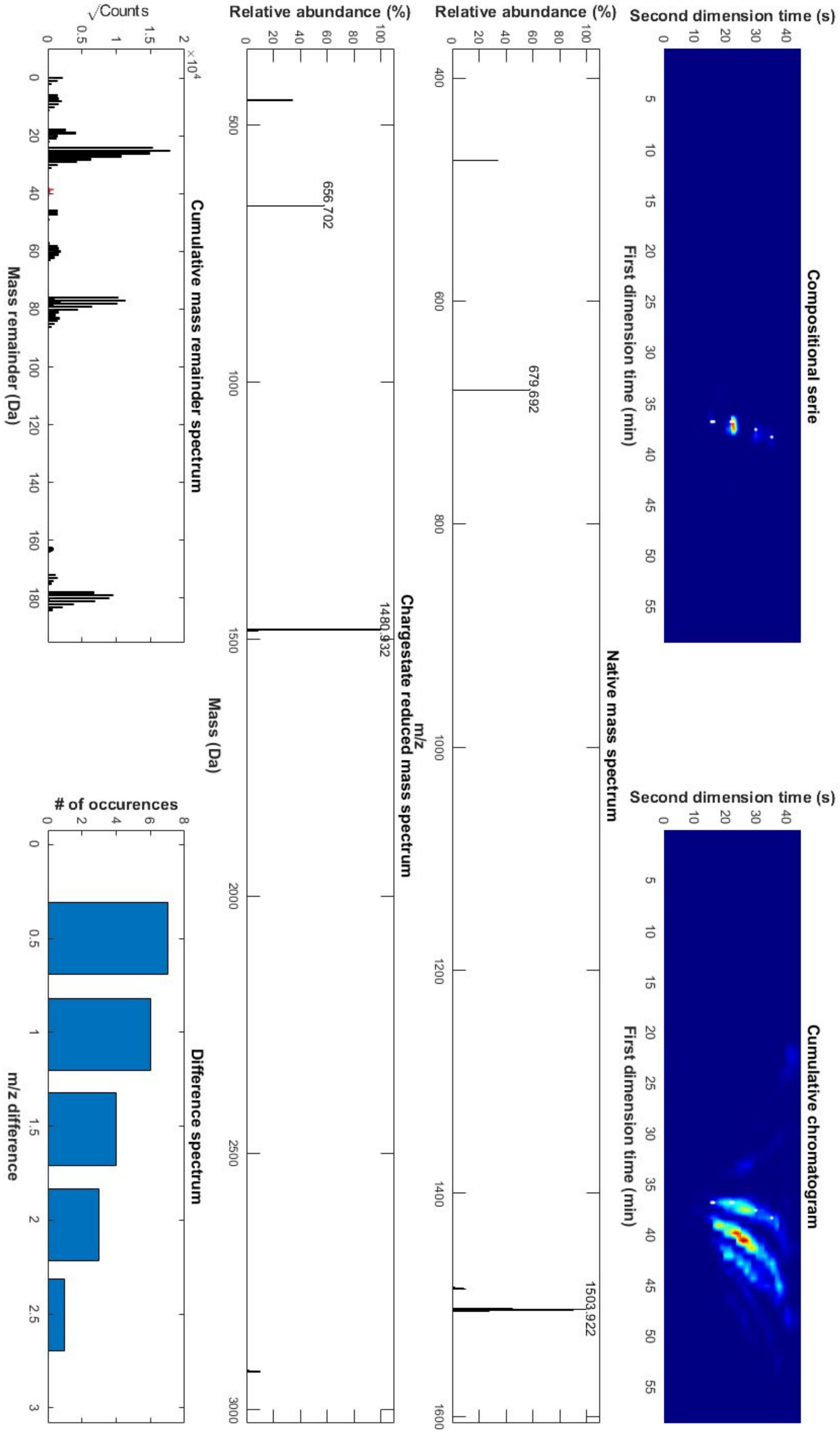

Figure S27: Wrongly assessed compositional series number 7. Charge-state reduction was poorly performed.

Wrongly assessed series 8 – Relative abundance: 1.5% –Incorrect charge-state reduction

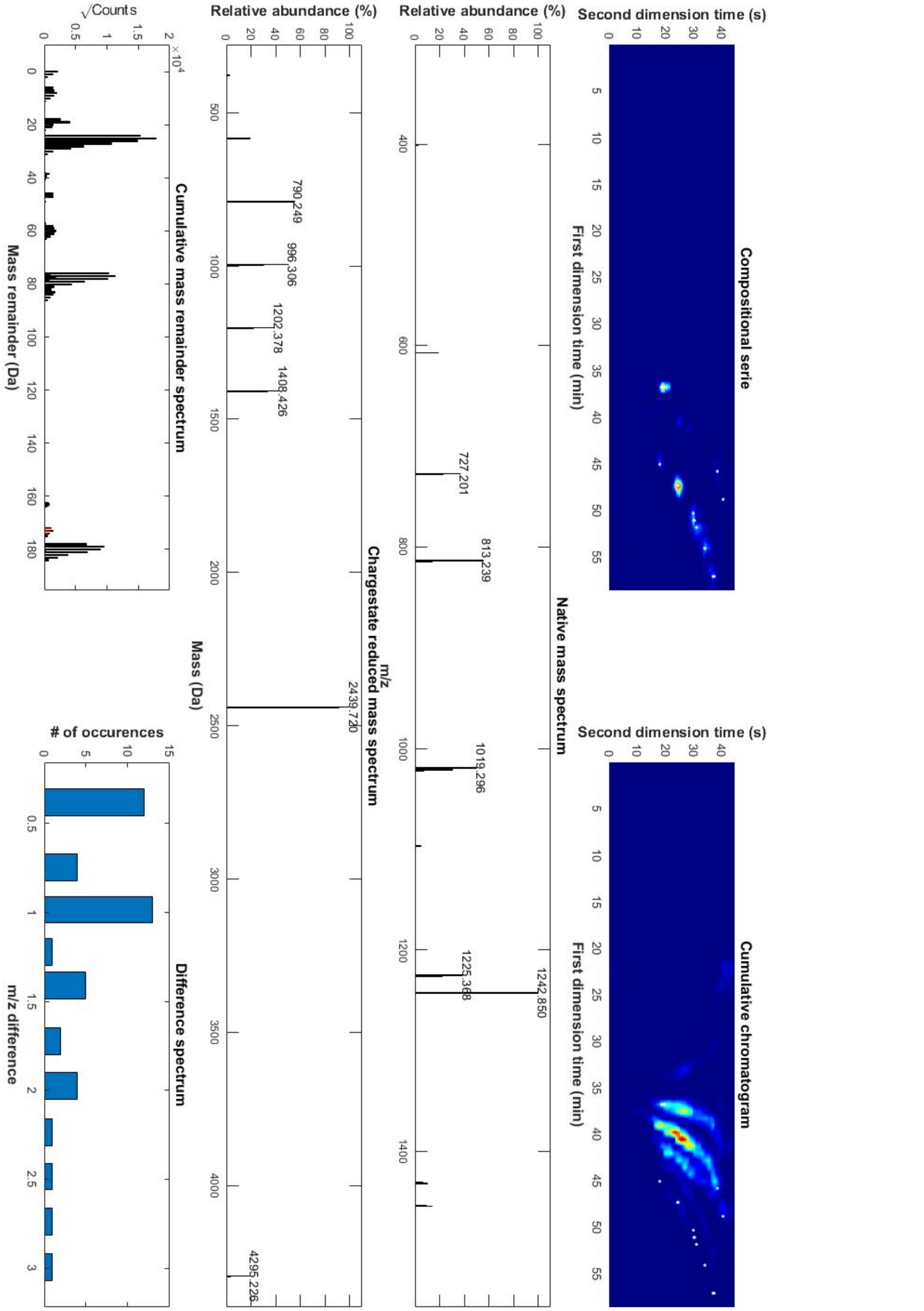

Figure S28: Wrongly assessed compositional series number 8. Charge-state reduction was poorly performed.

Compositional series 11 – Relative abundance: 1.4% – PG-PG + PG

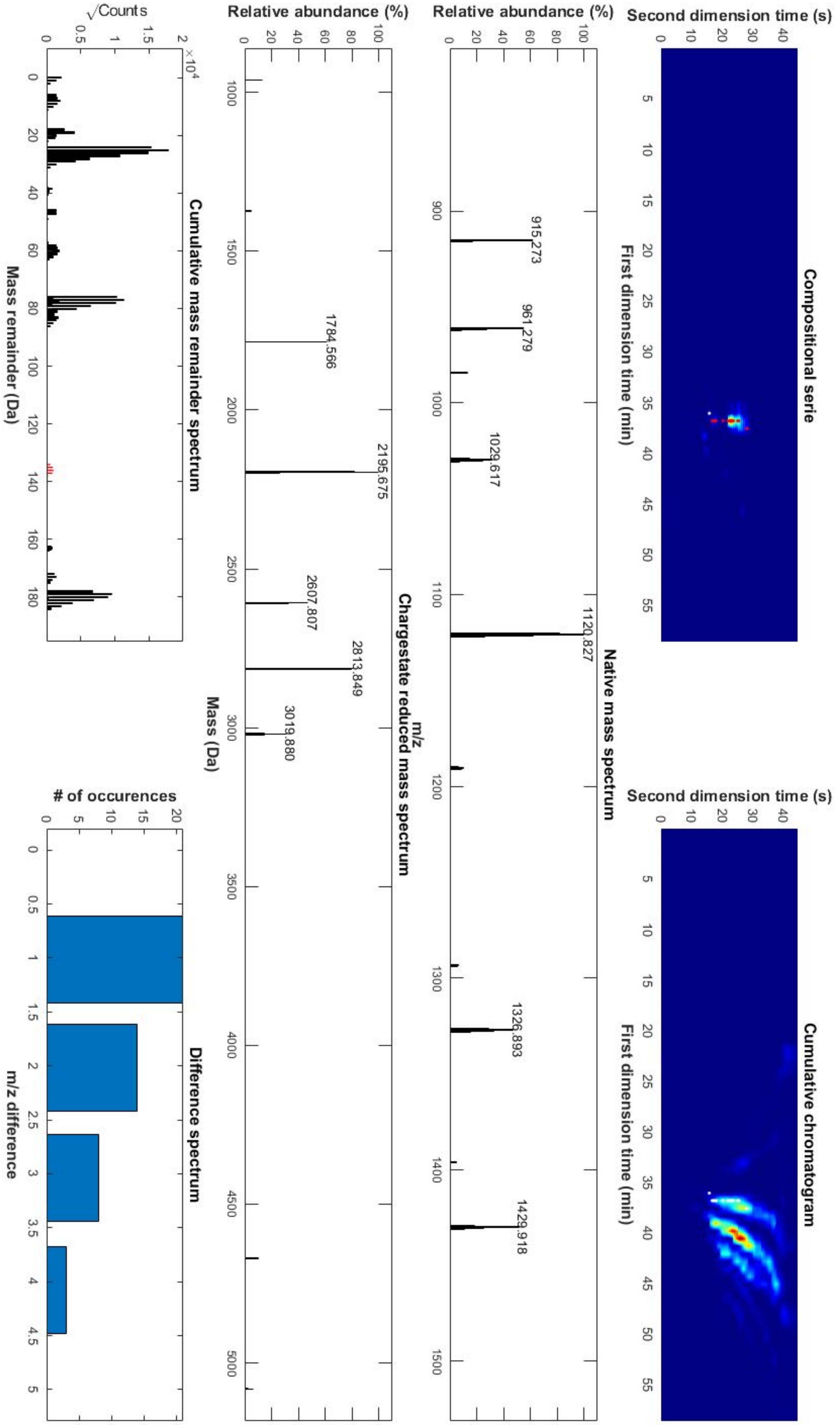

Figure S29: Compositional series number 11. Classified with end-groups PG-PG, but with an additional PG group within the polymeric chain.

Wrongly assessed series 9 – Relative abundance: 0.8% –Incorrect charge-state reduction

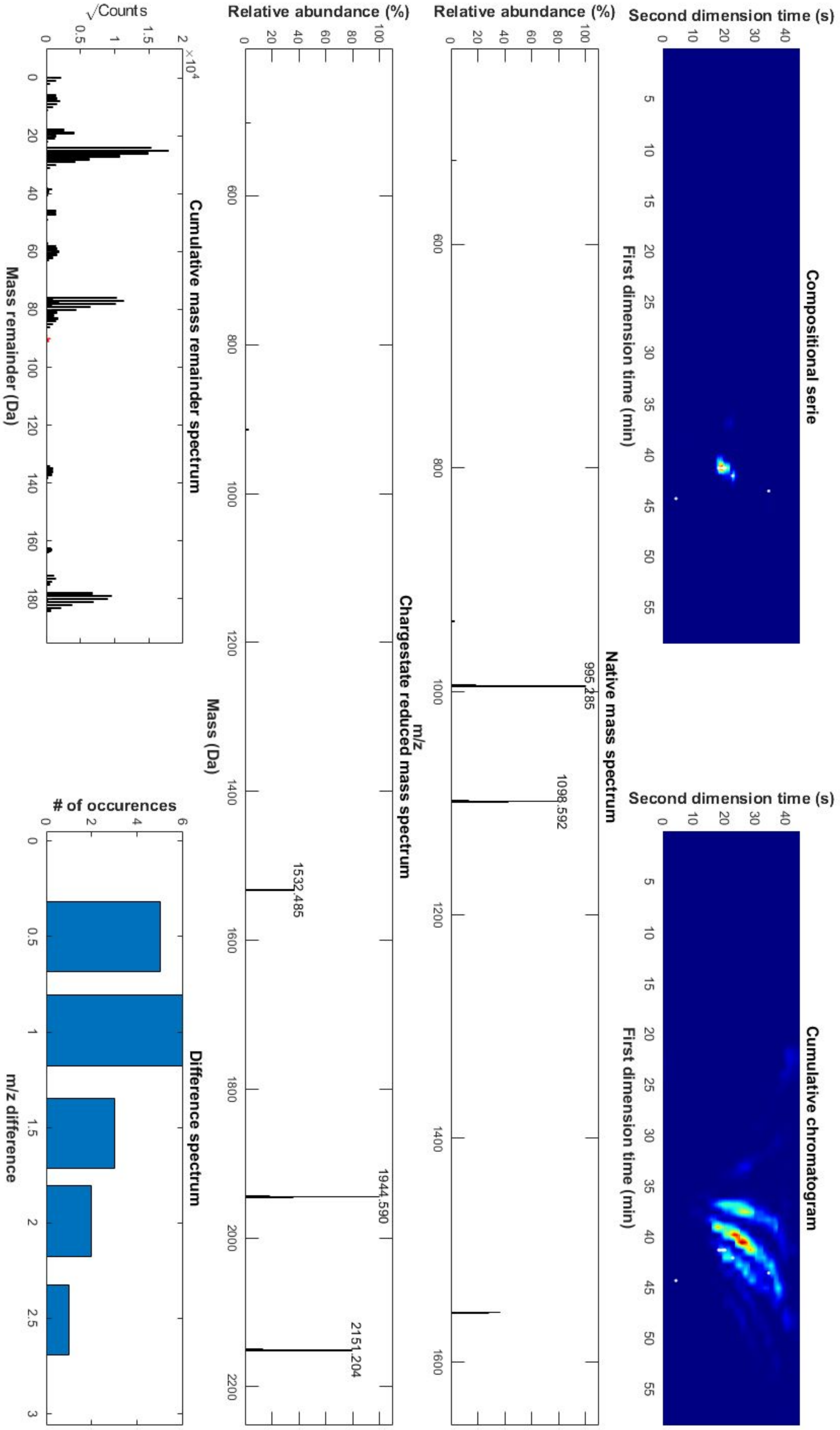

Figure S30: Wrongly assessed compositional series number 9. Charge-state reduction was poorly performed.

Wrongly assessed series 10 – Relative abundance: 0.8% –Incorrect charge-state reduction

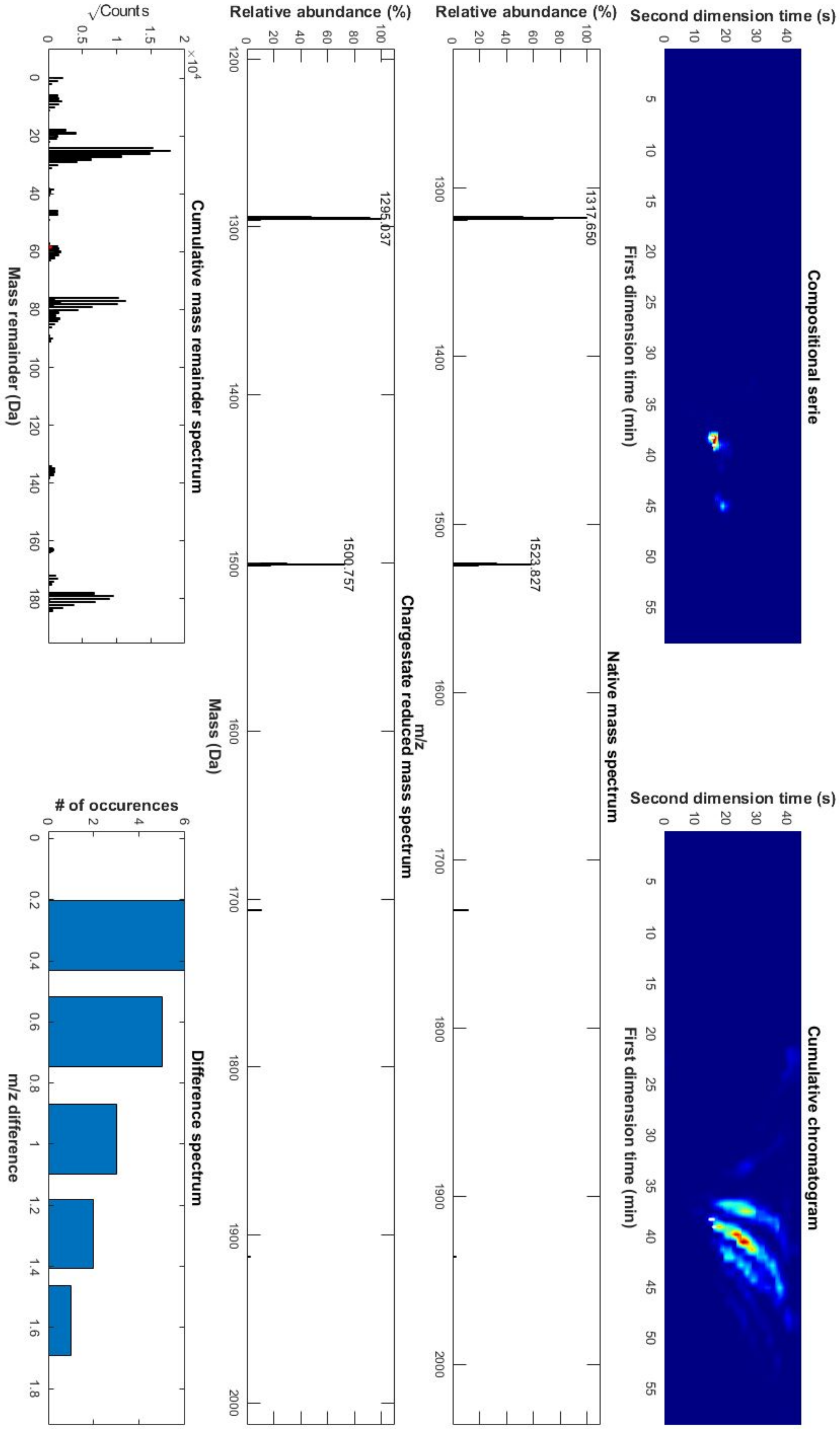

Figure S31: Wrongly assessed compositional series number 10. Charge-state reduction was poorly performed.

Wrongly assessed series 11 – Relative abundance: 0.8% –Incorrect charge-state reduction

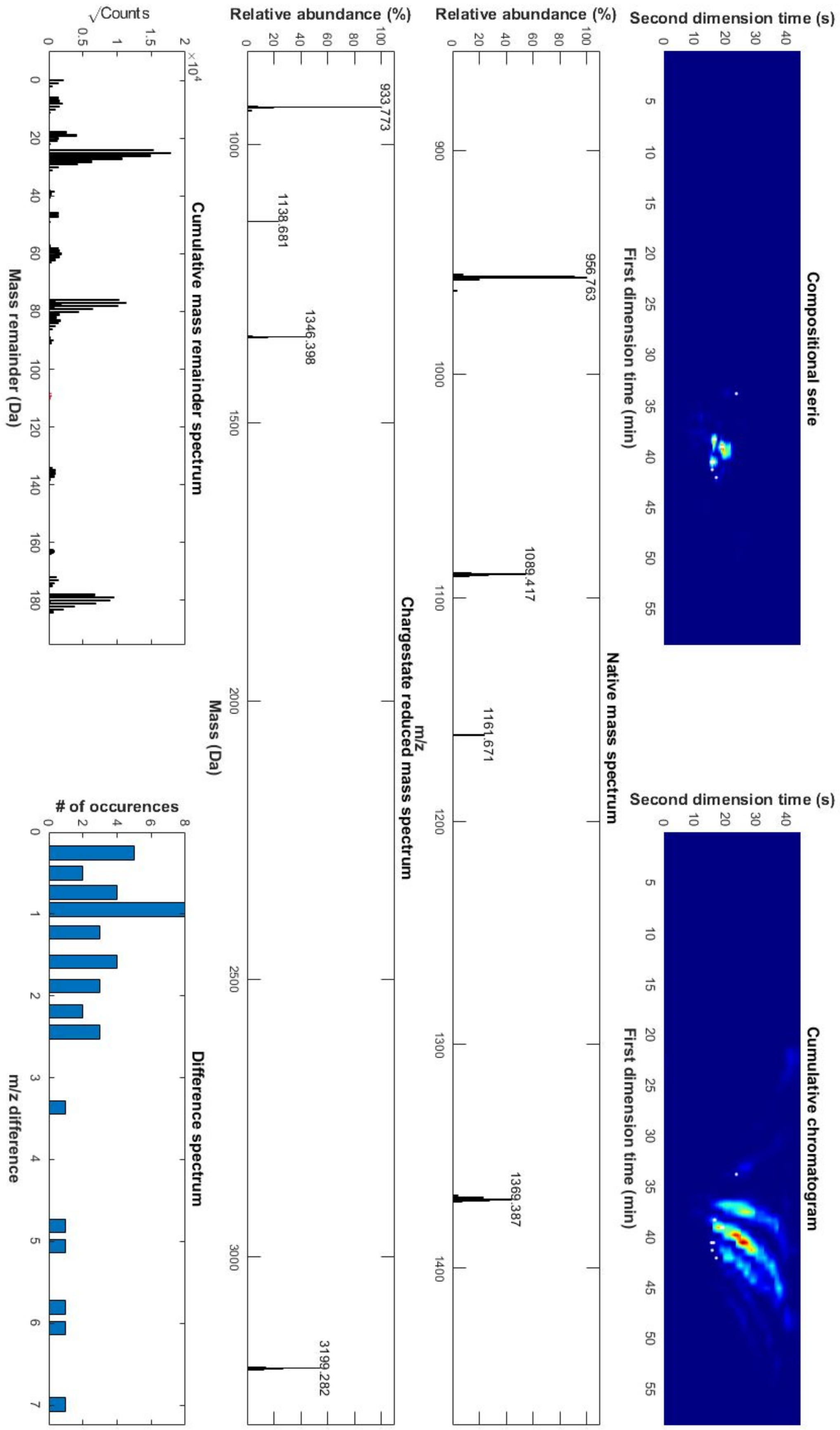

Figure S32: Wrongly assessed compositional series number 11. Charge-state reduction was poorly performed.

Wrongly assessed series 12 – Relative abundance: 0.8% – Incorrect charge-state reduction

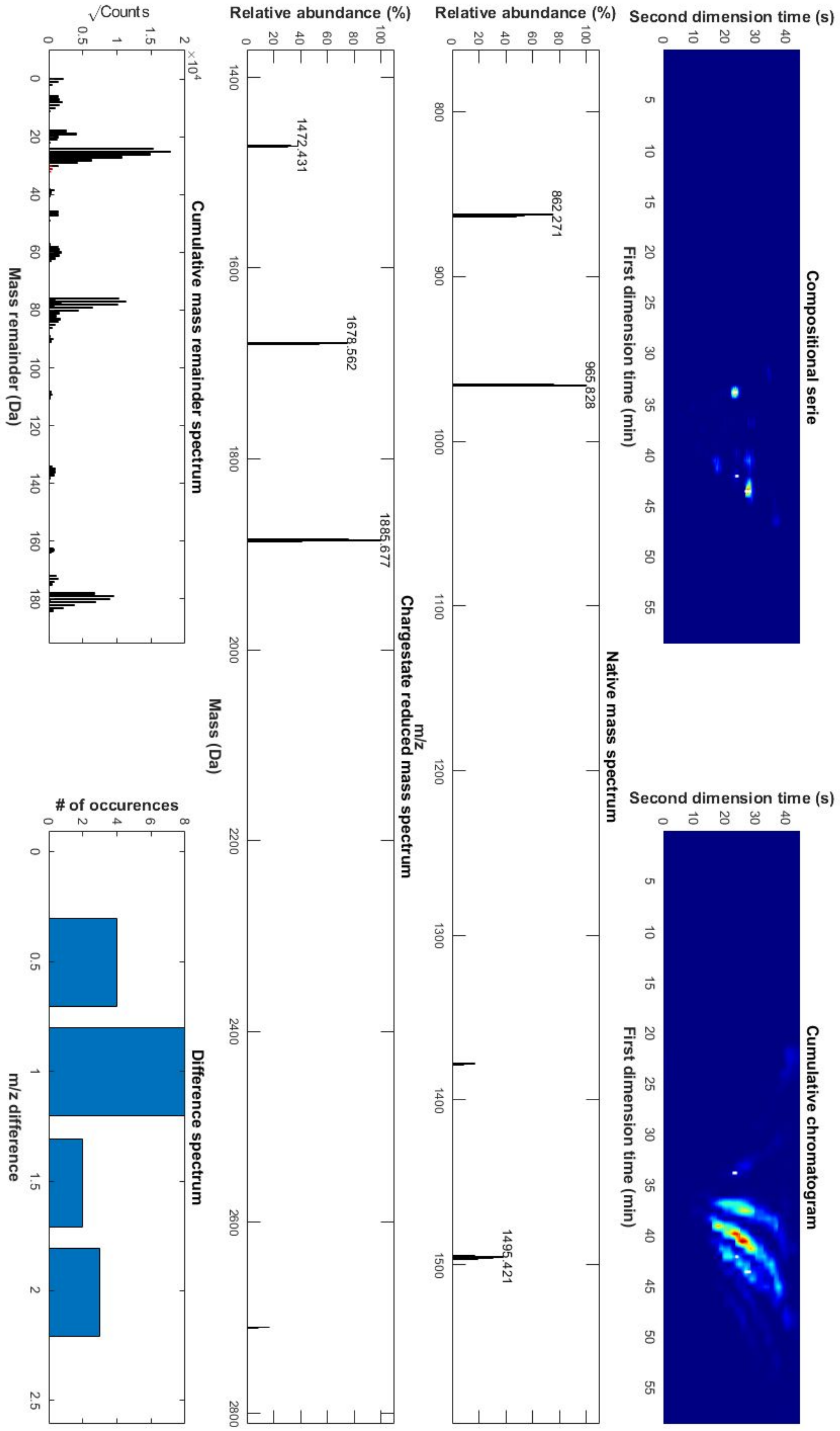

Figure S33: Wrongly assessed compositional series number 12. Charge-state reduction was poorly performed.

Wrongly assessed series 13 – Relative abundance: 0.7% –Incorrect charge-state reduction

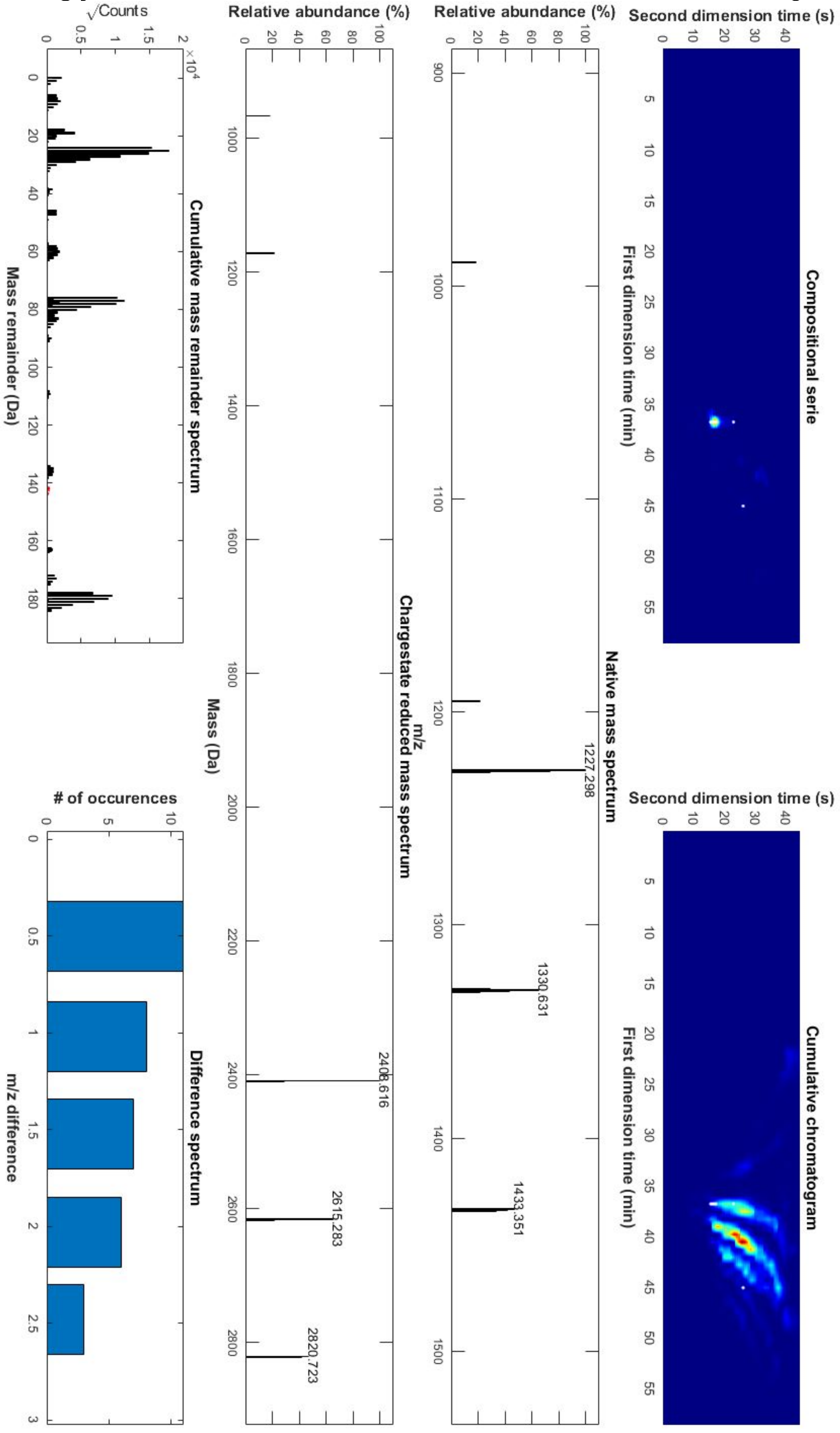

Figure S34: Wrongly assessed compositional series number 13. Charge-state reduction was poorly performed

Wrongly assessed series 14 – Relative abundance: 0.7% –Incorrect charge-state reduction

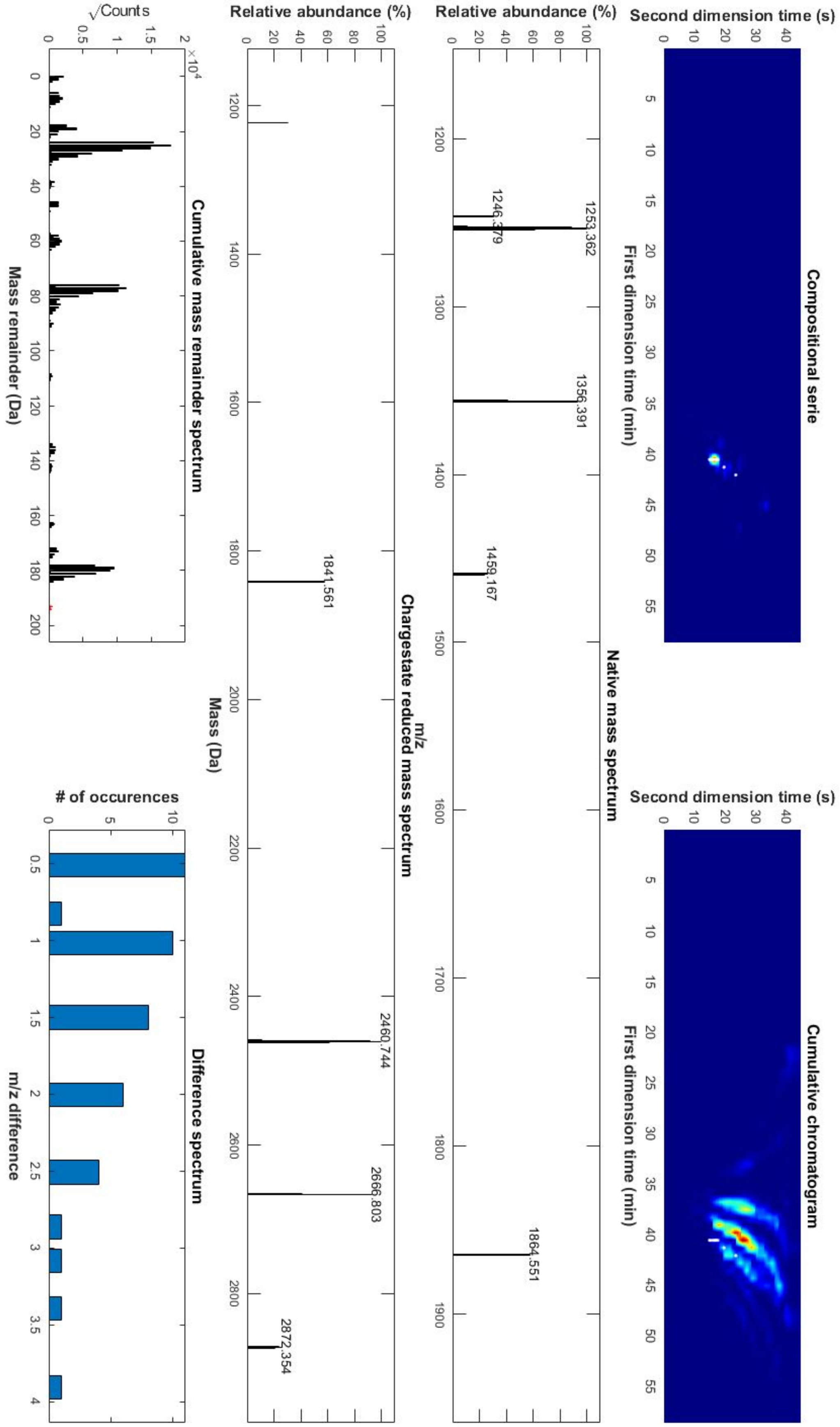

Figure S35: Wrongly assessed compositional series number 14. Charge-state reduction was poorly performed

Compositional series 12 – Relative abundance: 0.6% – [HHPA-HHPA]Na

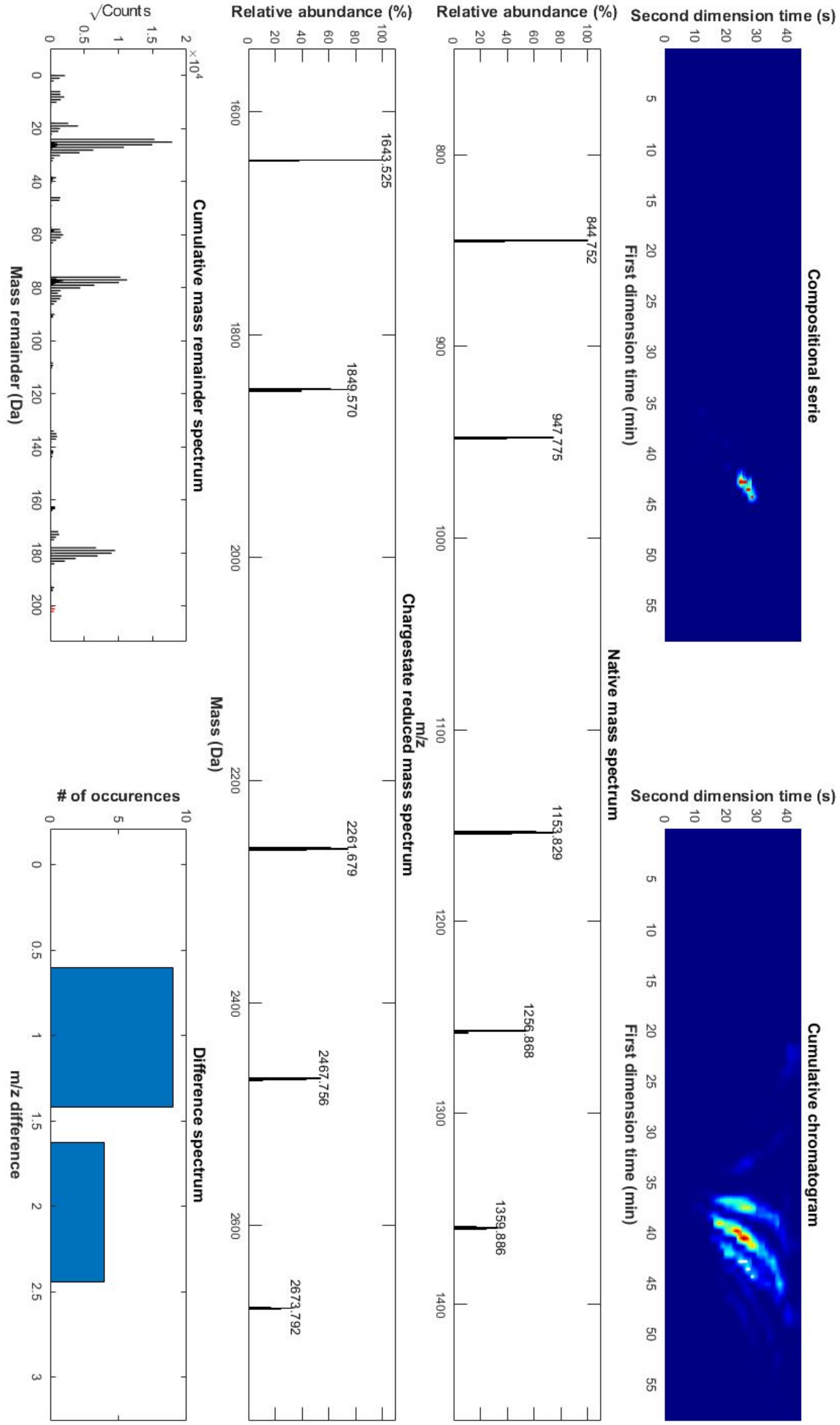

Figure S36: Compositional series number 12. Classified with end-groups HHPA-HHPA, but underwent sodium exchange within one of the free carboxylic acid of the cyclohexane dicarboxylic acid (Reaction product of the derivation with HHPA).
